# Proteomic Analysis of Morphologically Changed Tissues after Prolonged Dexamethasone Treatment

**DOI:** 10.3390/ijms20133122

**Published:** 2019-06-26

**Authors:** Abeer K. Malkawi, Afshan Masood, Zakia Shinwari, Minnie Jacob, Hicham Benabdelkamel, Goran Matic, Falah Almuhanna, Majed Dasouki, Ayodele A. Alaiya, Anas M. Abdel Rahman

**Affiliations:** 1 Department of Chemistry and Biochemistry, Concordia University, 7141 Sherbrook Street West, Montréal, QC H4B 1R6, Canada; 2 Department of Comparative Medicine, King Faisal Specialist Hospital and Research Center (KFSHRC), Riyadh 11461, Saudi Arabia; 3 Proteomics Resource Unit, Obesity Research Center, College of Medicine, King Saud University, P.O. Box 2925 (98), Riyadh 11461, Saudi Arabia; 4 Stem Cell & Tissue Re-Engineering Program, King Faisal Specialist Hospital and Research Center (KFSHRC), Riyadh 11461, Saudi Arabia; 5 Department of Genetics, King Faisal Specialist Hospital and Research Center (KFSHRC), Riyadh 11461, Saudi Arabia; 6 College of Public Health, Medical, and Veterinary Sciences/Molecular & Cell Biology, James Cook University, Townsville, QLD 4811, Australia; 7 College of Medicine, Al Faisal University, Riyadh 11533, Saudi Arabia; 8 Department of Chemistry, Memorial University of Newfoundland, St. John’s, NL A1B 3X7, Canada

**Keywords:** dexamethasone, label-free proteomics, LC-MS/M, rat tissues, glucocorticoid side effects, proteomic expression, network pathway

## Abstract

Prolonged dexamethasone (Dex) administration leads to serious adverse and decrease brain and heart size, muscular atrophy, hemorrhagic liver, and presence of kidney cysts. Herein, we used an untargeted proteomic approach using liquid chromatography-tandem mass spectrometry (LC-MS/MS) for simultaneous identification of changes in proteomes of the major organs in Sprague–Dawley (SD rats post Dex treatment. The comparative and quantitative proteomic analysis of the brain, heart, muscle, liver, and kidney tissues revealed differential expression of proteins (*n* = 190, 193, 39, 230, and 53, respectively) between Dex-treated and control rats. Functional network analysis using ingenuity pathway analysis (IPA revealed significant differences in regulation of metabolic pathways within the morphologically changed organs that related to: (i) brain—cell morphology, nervous system development, and function and neurological disease; (ii) heart—cellular development, cellular function and maintenance, connective tissue development and function; (iii) skeletal muscle—nucleic acid metabolism, and small molecule biochemical pathways; (iv) liver—lipid metabolism, small molecular biochemistry, and nucleic acid metabolism; and (v) kidney—drug metabolism, organism injury and abnormalities, and renal damage. Our study provides a comprehensive description of the organ-specific proteomic profilesand differentially altered biochemical pathways, after prolonged Dex treatement to understand the molecular basis for development of side effects.

## 1. Introduction

Dexamethasone (Dex) is an exogenous synthetic glucocorticoid (GC) with potent anti-inflammatory and immunosuppressive properties, which has made its use in clinics the mainstay for treatment of many inflammatory conditions, autoimmune diseases, and allergic reactions [1]. Glucocorticoids are known to regulate numerous physiological and metabolic processes [2] by genomic and non-genomic mechanisms [3] mediated through its binding to cytosolic glucocorticoid receptors (GRs). The GRs belong to a superfamily of ligand-regulated nuclear receptors that are widely expressed in the body and have gene targets of GRs signaling in a variety of tissues and cell types [4], including liver [5], adipocytes [6], and myotubes [7]. The presence of these widespread GRs allows the non-specific binding of Dex that result in the development of potentially harmful side effects that outweigh its benefits. Aside from this, the complexity of Dex action is increased with respect to its downstream signaling pathways, which vary differently among different tissues or organs, as well as within the same tissue but present at different locations. This differential action of Dex within the same organ was related to the differences in the tissue’s origin as well as to its microenvironment. Administration of Dex is known to preferentially increase central adiposity but also causes a decrease in the peripheral fat mass. It induces chondrogenesis of mesenchymal stem cells in the bone marrow while suppressing it in the synovium [8].

Long-term clinical use of Dex is discouraged as it leads to development of tissue-specific glucocorticoid resistance and serious side effects that include hypertension, diabetes, abdominal obesity and osteoporosis. The changes in the metabolite pattern in animal models treated with Dex were highlighted in our previous study that showed presence of hyperglycemia, weight change, osteoporosis, muscle atrophy, hemorrhage in the lung and liver [9], and kidney cysts [10]. These observed morphological changes might be the result of Dex-induced perturbations in the proteins related to the different metabolic and biochemical pathways in the different organs affected by its pharmacokinetics. Identification of this differential regulation of proteins and their involved pathways in different organs, either due to the direct or non-specific systemic binding, to the widely expressed GR, will help in furthering our understanding of the molecular actions of Dex. The morphogenic changes in the different organs, seen in our previous study, and identification of the cause of these changes will be of interest to explain the effects of prolonged Dex use. Single protein datasets from individual organs provide only lists of proteins. On the other hand, integration of the proteins from the different organs, at the same time, will provide a better picture of complete biochemical changes and protein dynamics taking place within the animals [11].

Proteomic techniques have become the mainstay in providing insights into the mechanisms of the biological process through characterization of cellular protein composition and functional linkages between protein molecules. Previous studies have used the proteomic approach to study changes in different rat tissues only one at a time, such as lens crystallins [12,13], retina [14], bone marrow mesenchymal stem cells [15], heart [16], and liver [17]. In a recent study, Biancotto et al. [18] identified proteins altered in bovine liver after Dex treatment by using an untargeted shot-gun proteomics approach based on tandem mass tags. The untargeted proteomic analysis provides an assessment tool to assess the metabolism and the adverse effects of the drug, which cannot be evaluated using classical drug assays that are limited to single molecules or targeted towards single pathways. Comprehensive biochemical profiling and characterization of these proteome-wide alterations in different tissues will enable us to understand the complex interacting metabolic events that occur within a cell, its pathophysiology, and determine signature proteins [19]. Recently, Hinkelbein et al. [20] carried out a simulataneous proteomic profiling of the rat organs to identify the affected pathways after short-term hypoxia. In the present study, we used a label-free quantitative proteomics-based liquid chromatography-tandem mass spectrometry (LC-MS/MS) approach to simultaneously study the unique protein signatures in different rat tissues after long-term exposure to Dex treatment as a snapshot in time. 

## 2. Results

### 2.1. Clinical Phenotypes

**Figure 1:**
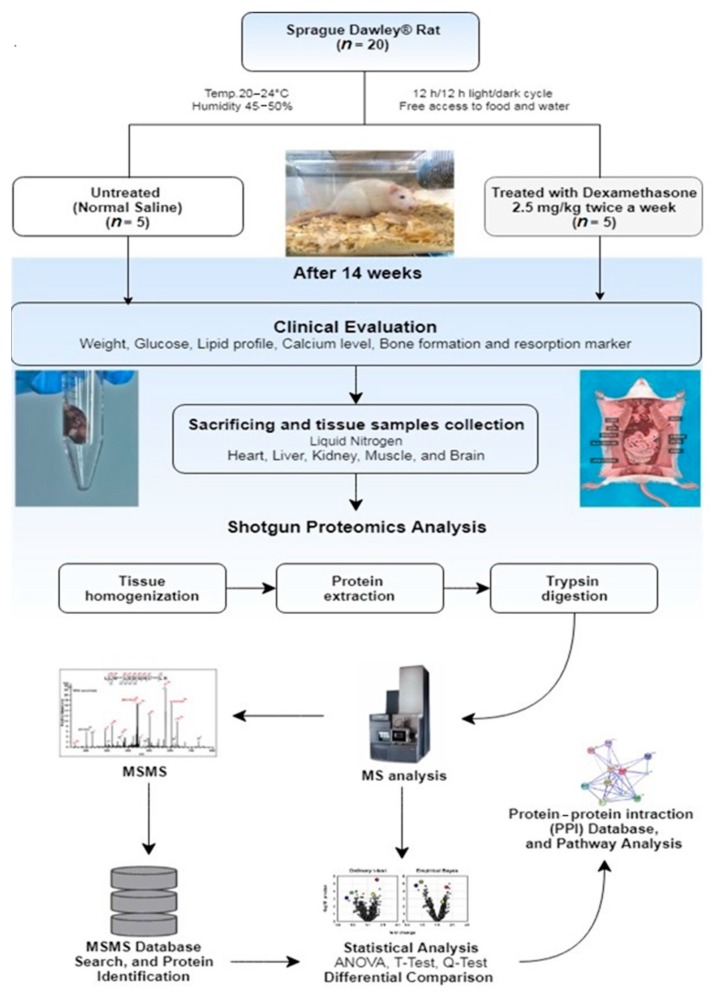
A flow chart of the animal study design and proteomics based liquid chromatography-tandem mass spectrometry (LC-MS/MS) experiment. Sprague–Dawley rats were divided into two groups; control (*n* = 10) and Dex (*n* = 10). After 14 weeks of twice-weekly treatment with Dex, the proteomics profile of freshly collected tissue samples from major body organs were studied using shotgun proteomics analysis.

During the experiment, the Dex-treated rats went through several phenotypic changes such as reduction in age-dependent body weight by ~20%, elevation in blood glucose and triglyceride (TG) levels with markedly reduced low-density lipoprotein in comparisonto the control group (*p* < 0.001), as reported previously [10]. During the sacrificing of the animals, distinct morphological changes in the soft-tissue mass and variation in organ size in the Dex-treated animals were noted [10]. Both the brain and the heart were determined to be smaller in size, the skeletal muscle showed atrophy, the liver demonstrated grossly hemorrhagic changes, and cystic changes of the kidney were noted in the Dex-treated animals as compared to the control group. A triplicate set of samples were taken for proteomics analysis from the same pool from each organ as detailed in Figure 1. 

**Figure 2:**
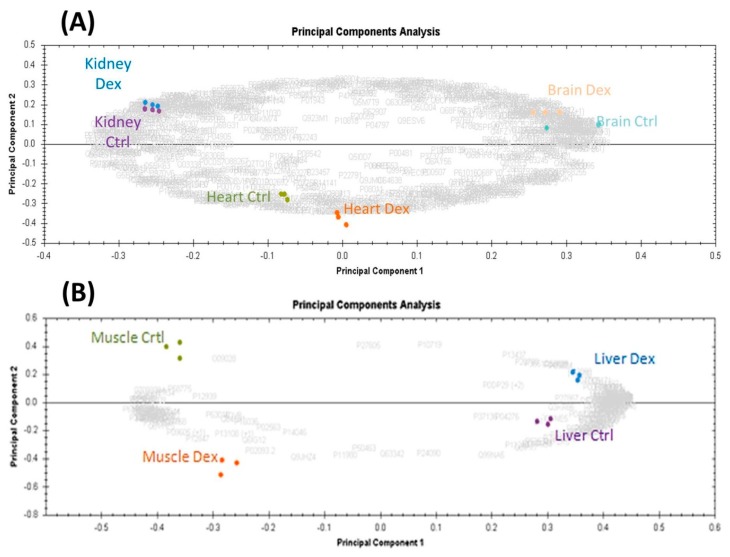
(**A**) Principal component analysis (PCA) plot of brain, heart, and kidney tissue samples using the protein expression dataset. Shown are the brain (orange, blue), heart (orange, green), and kidney (violet, purple) tissues between Dex-treated and control rats, respectively. (**B**) PCA plot of liver (blue, purple) and muscle (orange, green) tissues between Dex-treated and control rats, respectively. The separation seen between the treated and the control groups in each tissue sample represents the biological differences due to the prolonged Dex treatment. The letters in grey color in the background represent the accession numbers of all the implicated proteins in the analysis. (Data were generated using Progenesis QI for proteomics (ProgenesisQIfp version 2.0.5387, Nonlinear Dynamics/Waters.).

The protein expression datasets obtained after LC MS/MS mass spectrometric analysis, in samples from each of the individual organs, were compared by running a principal component analysis (PCA) analysis. The PCA plots constructed from them showed a clear separation between the control and Dex-treated samples for the brain, kidney, heart, and muscle samples (Figure 2A) and skeletal muscles and liver (Figure 2B).

**Figure 3:**
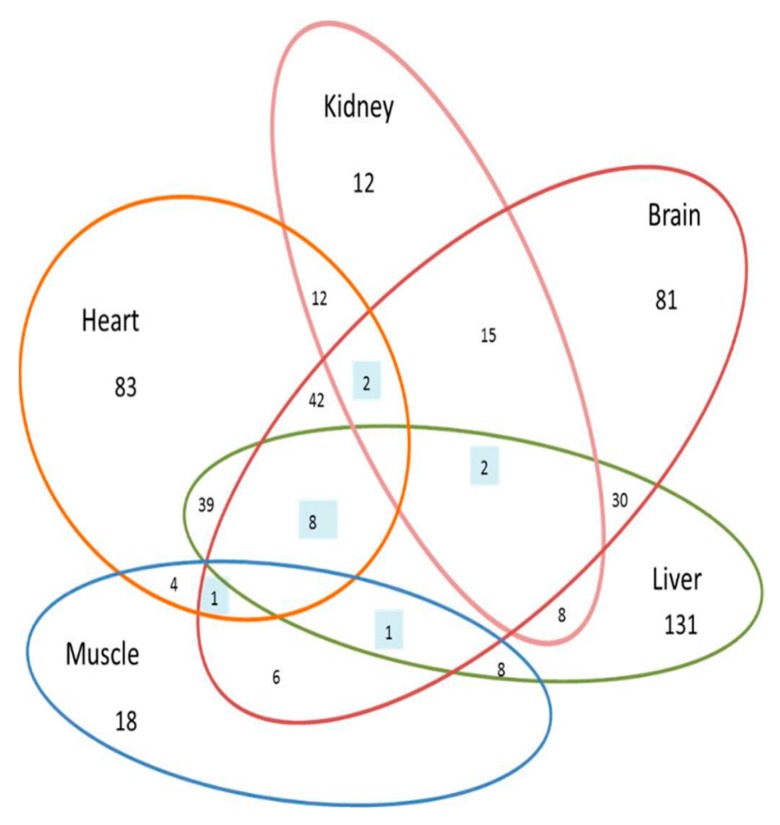
Organ-specific proteome expression: Venn diagram representation of the differentially expressed significant proteins (*p* ≤ 0.05, 1.5 fold change) identified within the morphologically altered organs from Dex-treated and control animals. Number of proteins expressed in common among various organs are shown in the intersections. The number of proteins specific for each organ namely the heart (*n* = 83), kidney (*n* = 12), brain (*n* = 81), liver (*n* = 131), and muscle (*n* = 18). The highest number of overlaps was found to be between the heart and brain (*n* = 42) followed by the heart and liver (*n* = 39) and the liver and brain (*f* = 30), while only eight proteins were common among the three groups and no proteins were in common between the muscle and kidney. The area of the Venn diagram is not representative of the numbers of identified proteins.

The separation between the Dex-treated and control samples in different tissues was apparent in all the morphologically altered organs, where the cutoff values to filter these proteins were *p*-values < 0.05, and fold change > 1.5. The number of relatively expressed proteins identified between the Dex-treated group compared to the control in the five different organs and the proteins in common between them are depicted in the Venn diagram (Table 1, Figure 3).

### 2.2. Mass Spectromeric Protein Identification and Analyses 

A label-free MS-based tool was used for quantitative and comparative expression analysis of the protein changes between the prolonged Dex-treated versus control rats in different organs. The proteomic analysis revealed 190 significantly differentially expressed proteins (ANOVA test, *p* < 0.05, >1.5 fold change (FC)) in the brain (91 up and 99 down), 193 proteins in the heart (78 up and 115 down), 39 proteins in the muscle (30 up and 9 down), 230 protein in the liver (128 up and 102 down), and 53 proteins in the kidney (38 up and 15 down) in the Dex-treated versus control rats. A list of all the differentially expressed proteins identified in the different organs with their fold changes is shown in Supplementary Materials Table S1. The detailed table of the list of proteins with their mass spectrometry data, mean abundances, and standard deviations can be accessed at the peptide atlas database [21].

**Figure 4:**
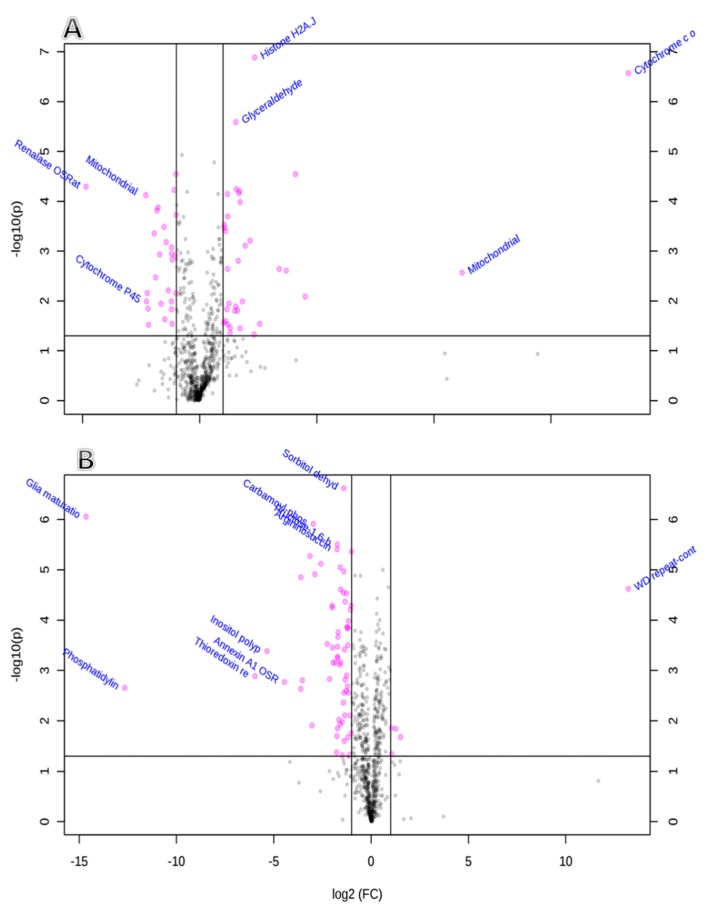

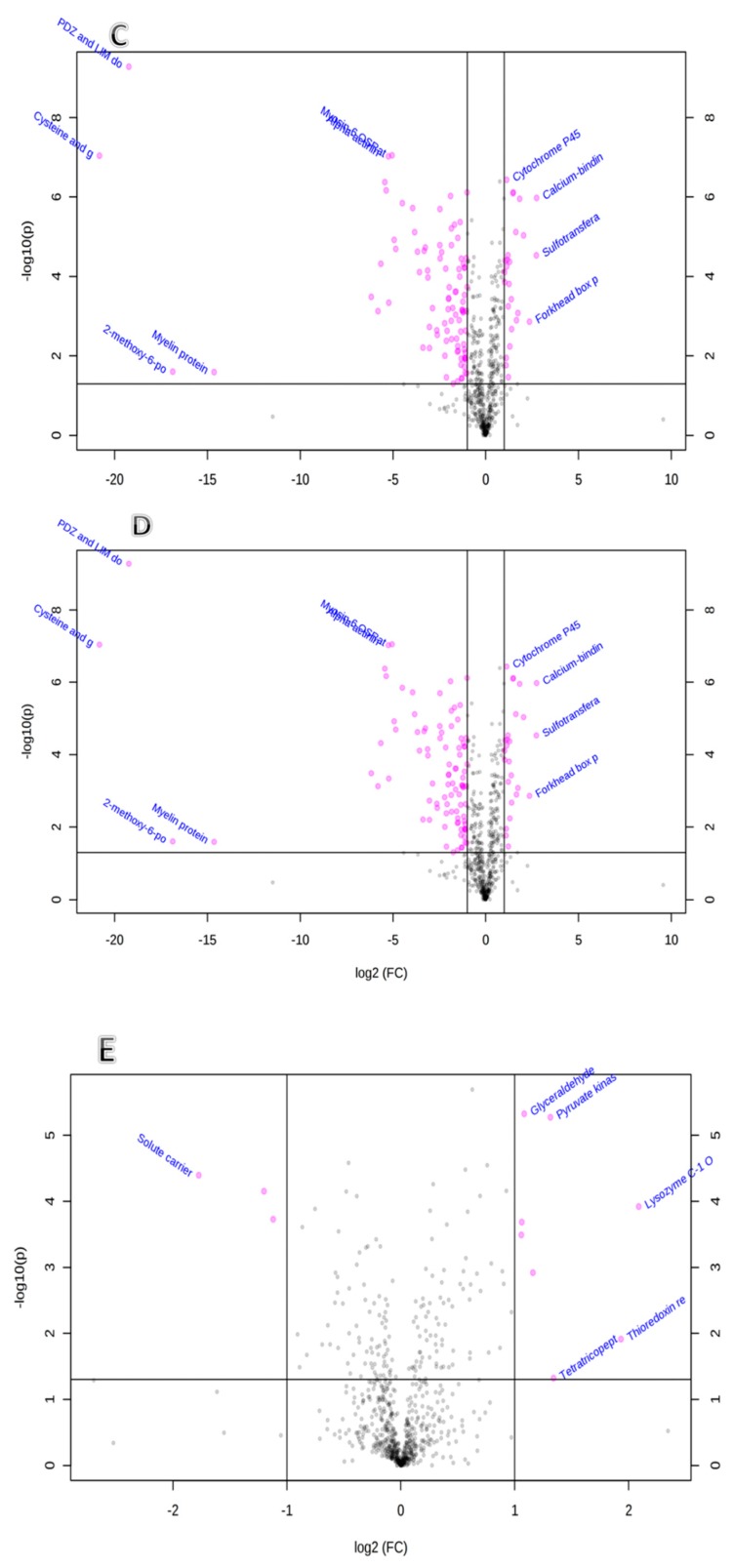
Plots showing the statistically significant expressed proteins in brain tissue (**A**), heart (**B**), skeletal muscle (**C**), liver (**D**), and kidney (**E**) after prolonged Dex administration false discovery rate (FDR-corrected *p*-value < 0.05), and fold change FC > 1.5 were visualized in each volcano plot in the left and right corners as down- and upregulated, respectively, in the Dex-treated rats.

We further analyzed the identified proteins statistically using the *t*-test and by applying false discovery rate (FDR)-corrected *p*-values (*y*-axis), and fold change (FC) (*x*-axis) analyses were evaluated and visualized graphically using the volcano plot. The significant features shown in the volcano plot in the brain (Figure 4A), heart (Figure 4B), liver (Figure 4C), muscle (Figure 4C), and kidney (Figure 4D) are the ones that passed the FC and FDR-corrected *p*-value thresholds of 1.5 and 0.05, respectively. Proteins shown in pink dots in the upper right and left corners of the plot, respectively, represent significantlydownregulated or upregulated features upon Dex treatment. 

The differentially abundant protein sets identified from the brain, heart, muscle, liver, and kidney tissues were next uploaded and investigated further using the ingenuity pathway analysis (IPA) software to determine their functional and biological roles.

#### 2.2.1. Gene Ontology and Functional Analysis of the Identified Proteins in the Five Organs

Gene ontology annotation and functional analysis of the identified proteins were carried out to ascertain the different types of identified proteins and major biochemical functions associated with them that were affected after prolonged Dex treatment. The significantly differentially abundant proteins in the different rat organs were classified according to their function and location. Proteins significantly enriched in the brain, heart, muscle, liver, and kidney were classified by the IPA as enzymes (*n* = 82, 104, 21, 126, 20, respectively), ion channel proteins (*n* = 4, 1, 1, 3, 0, respectively), kinase (*n* = 9, 7, 0, 8, 2, respectively), transcription and translation regulators (*n* = 8, 5, 2, 11, 0, respectively), transporter (*n* = 22, 19, 3, 20, 7, respectively), and others (*n* = 65, 57, 12, 62, 24, respectively). In terms of the location of the identified proteins in the brain, heart, muscle, liver, and kidney, in that order, they were localised by the IPA, mainly to the cytoplasm (*n* = 120, 137,27, 181, 32, respectively), plasma membrane (*n* = 22, 22,4, 15, 6, respectively), nucleus (*n* = 21, 19, 1, 6, 2, respectively), extracellular space (*n* = 16, 10, 2, 12, 9, respectively), and others (*n* = 6, 5, 5, 6, 4, respectively). 

The molecular and functional processes affected by Dex treatment revealed the involvement of different biological processes within these organs. The two top significantly altered molecular functions (and number of associated proteins) that were identified in the brain were related to cellular compromise (*n* = 42) and molecular transport (*n* = 65); in the heart and liver they were related to amino acid metabolism (*n* = 21, 26, respectively) and small molecule biochemistry (*n* = 86, 103, respectively). In the muscle, these processes related to vitamin and mineral metabolism (*n* = 5) and energy production (*n* = 5), and in the kidney, they were related to drug metabolism (*n* = 5) and molecular transport (*n* = 16) (Supplementary Materials Table S3). Dex treatment was also found to affect and regulate the canonical pathways differently in each organ. The top canonical pathway affected in the brain post-prolonged Dex treatment was found to be the leucine degradation pathway, while that in the muscle was glutathione redox reactions. The top canonical function affected in the kidney, liver, and heart was found to be LPS/IL-1-mediated inhibition of LXR and RXR function. This is in line with previous studies that demonstrated glucocorticoid-mediated regulation of this pathway to affect lipid metabolism [22] (Supplementary Materials Table S4).

#### 2.2.2. Network and Pathway Analysis

The protein expression datasets identified in the different organs were uploaded into the IPA software to identify the pathways related to these proteins. The networks generated by this approach are preferentially enriched for proteins with the most extensive and specific interactions using the ingenuity knowledge base. The interacting proteins are represented as nodes, the direct biological relationship among two nodes as a line and an indirect relationship as a hashed line. 

**Figure 5:**
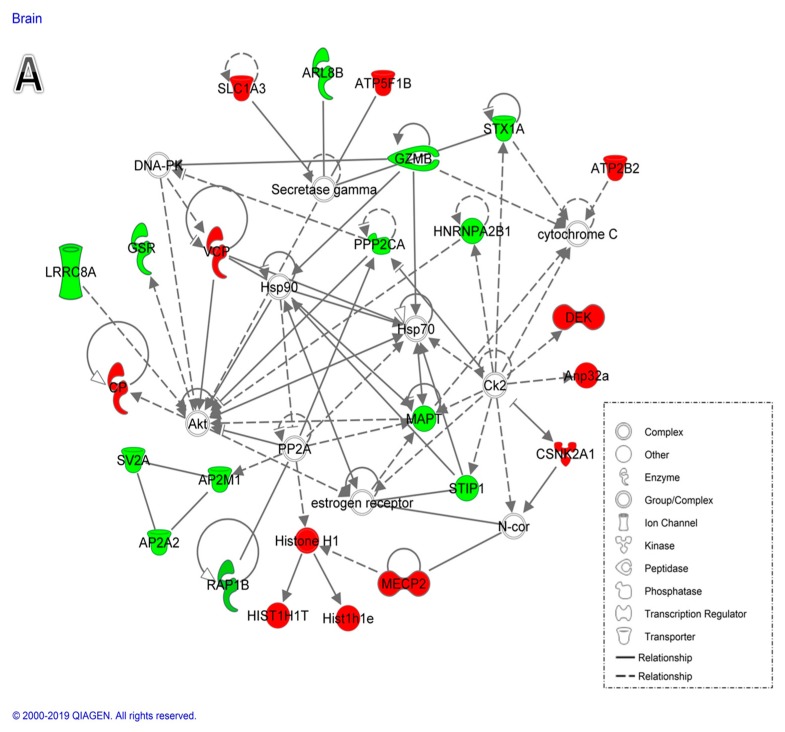

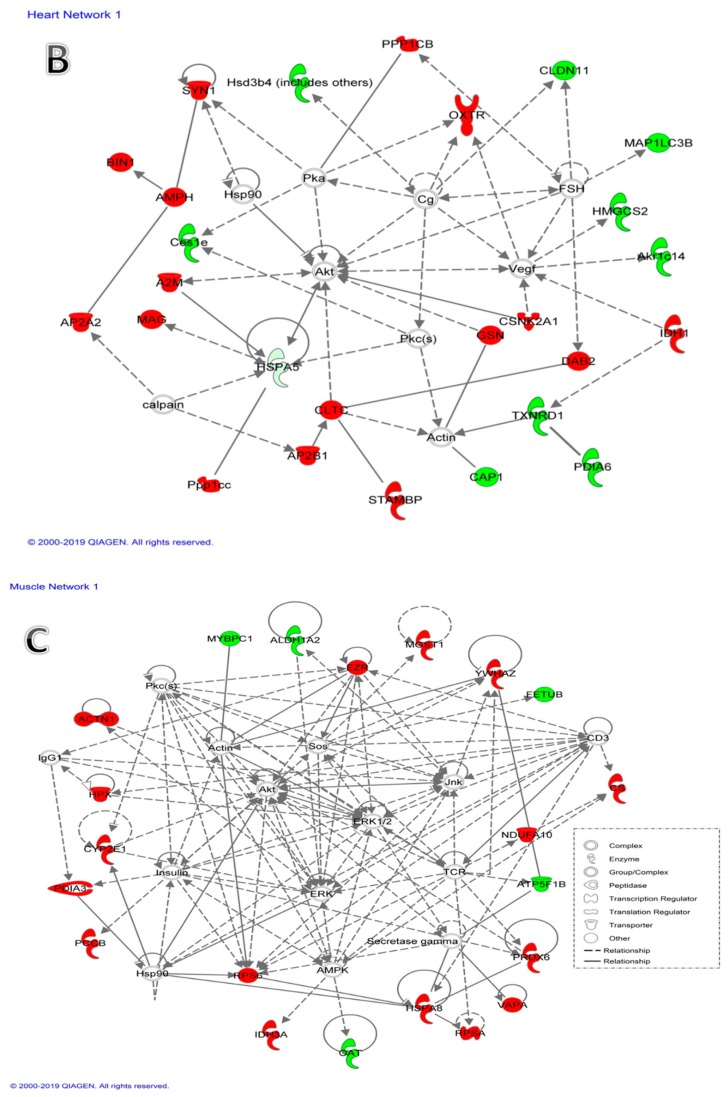

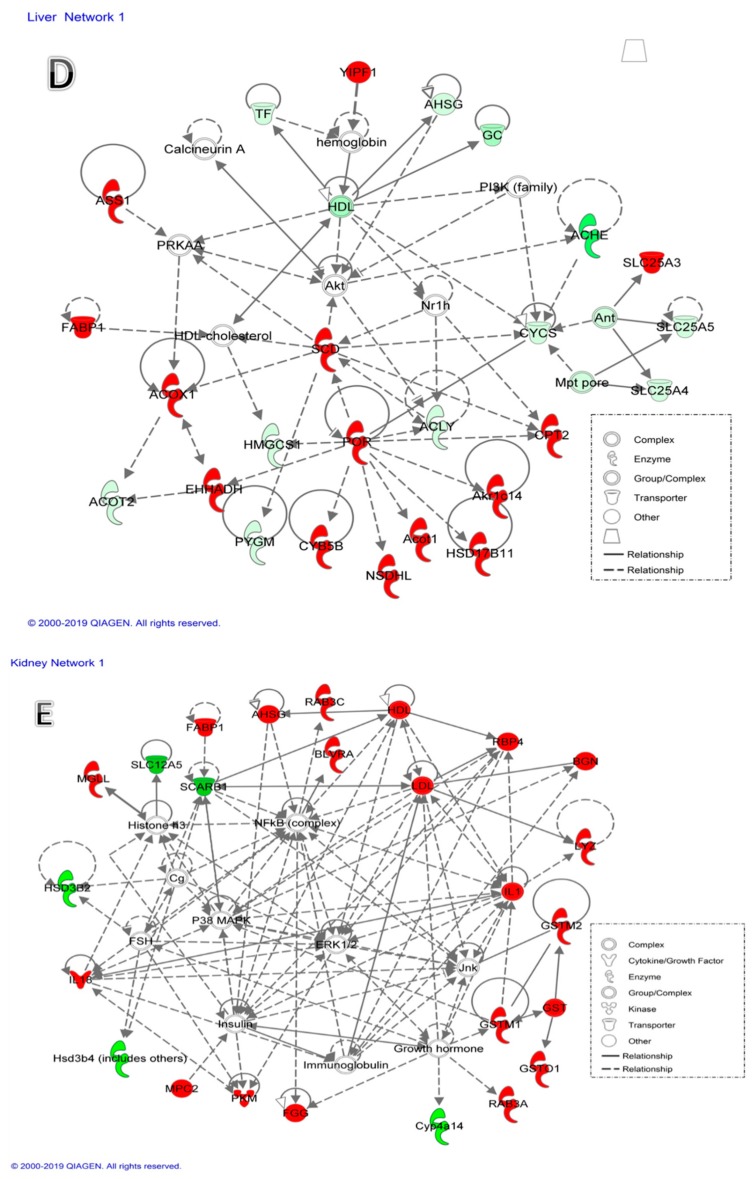
Network analysis of the differentially expressed proteins between the Dex-treated and control rats identified by the IPA (ingenuity pathway analysis). (**A**) Brain network pathway converged on the central signaling pathway involving extracellular signal–regulated kinases (ERK1/2), janus kinase (Jnk), and Akt. The network with the highest score related to cell morphology, nervous system development, and function and neurological disease (score = 38). (**B**) Heart network pathway identified the involvement of Akt and phosphoinositide 3-kinase (PI3 kinase) signaling pathways. The network with the highest score related to cellular development, cellular function and maintenance, and connective tissue development and function (score = 43). (**C**) Skeletal muscle network pathway identified with the highest scoring network (score = 50) related to nucleic acid metabolism, small molecule biochemistry, and vitamins and mineral metabolism. (**D**) Liver network pathway was identified with the highest scoring network (score = 41) related to lipid metabolism, small molecule biochemistry, and nucleic acid metabolism. (**E**) Kidney network pathway was identified with the highest scoring network (score = 41) related to drug metabolism, organism injury, and abnormalities and renal damage. Nodes in green and red correspond to down- and upregulated proteins, respectively. Non-colored nodes were proposed by IPA and suggest potential targets functionally coordinated with the differential proteins. Solid lines indicate direct molecular interactions and dashed lines represent indirect relationships.

Network analysis of the differentially expressed proteins between the Dex-treated and control rats in the brain identified the pathway with the highest score, related to cell morphology, nervous system development, and function and neurological disease (score = 38) (Figure 5A). The central node of the identified pathway was mitogen–activated protein (MAP) kinase whose activity was suggested to be reduced in the Dex-controlled rats versus controls. In the heart tissue, the pathway identified was related to cellular development, cellular function and maintenance, and connective tissue development and function (score = 43) (Figure 5B) with the protein kinase B (Akt) as its central node. The pathway identified in the muscle with the highest score related to nucleic acid metabolism, small molecule biochemistry, and vitamins and mineral metabolism (score = 50) (Figure 5C), and identified janus kinases (Jnk), protein kinase C, insulin, and extracellular-signal regulated kinases ERK as the central nodes involved in the regulation of Dex action. The highest scoring network pathway identified in the rat liver related to lipid metabolism, small molecular biochemistry, and nucleic acid metabolism (score = 41) (Figure 5D), and identified Akt and phosphoinositide 3-kinase (PI3 kinase) as the central nodes. In the kidney, the pathway identified with the highest score was related to drug metabolism, organism injury, and abnormalities and renal damage (score = 41) (Figure 5E), and showed Jnk, ERK, insulin, nuclear factor kappa-light-chain-enhancer of activated B cells NFkB, and p38 MAP kinases as the central nodes. 

## 3. Discussion

The synthetic GC derivative Dex mimics the actions of natural glucocorticosteroids and affects nearly all the tissues and physiological processes that are regulated by them. The effectiveness of long-term therapy with Dex is limited due to the serious side effects that negate its clinical benefits. These effects, for the most part, are mediated via the genomic and non-genomic signaling pathways [23] through interactions with the pleiotropic GR, which is expressed in nearly every cell of the body [24]. The GRs have variable cell-type-specific effects on proliferation, differentiation, and apoptosis and are maintained as an inactive cytoplasmic complex with cofactors, such as heat-shock proteins and immunophilins [25]. A holistic understanding of the diverse cellular responses and heterogeneity in GR signaling in diseased tissues will aid in understanding the side effects that arise due to their pharmacological use. Numerous studies have been undertaken in the past to elucidate the mechanism of developments of Dex-related adverse reactions. However, even with this bulk of literature, the exact mechanism of the overall systemic Dex action has not yet been elucidated.

A majority of the previous studies used a targeted approach focusing on either a single pathway or a target organ to identify the mechanisms of development of adverse effects. In the present study, we used an untargetted LC-MS/MS quantitative proteomic approach to analyze the proteome of the morphologically altered individual organs (i.e., brain, heart, muscle, liver, and kidney) between the controlled and the Dex-treated SD rat model, after development of side effects. The Dex-treated male rats were found to suffer from a severe reduction in weight gain, high blood sugar, essential changes in serum lipid, and reduction in total serum alkaline phosphatase (ALP) [10]. The animal model provided us with an effective method for profiling the differences in protein expression within each tissue and also among them to allow identification of organ-specific Dex-related adverse effects [26]. 

### 3.1. Proteomic Profiling of the Major Tissues Affected by Dex Treatment

The proteomic profiling of the individual protein datasets, from the five different morphologically altered organs, identified the involvement of not only different proteins but also the number of proteins. The highest number of significantly altered proteins, between the Dex-treated and control rats, were found in the dataset of the liver (*n* = 230) followed by the heart (*n* = 193), brain (*n* = 190), kidney (*n* = 53), and the muscle (*n* = 39). 

#### 3.1.1. Proteins Related to the Reduced Brain Size after Dex Treatment

The action of Dex and glucocorticoids are known to affect the brain and the central nervous system, causing psychiatric and cognitive–behavioral and memory disturbances [27]. In their proteomic study, Feldman et al. [28] demonstrated that increased corticosterone levels mimic chronic stress and were associated with hippocampal atrophy and cognitive dysfunction. We found that the pathways in the rat brain related to cell morphology, nervous system development and function, and neurological disease with the central nodes focused around tau protein, Akt, and PI3K signaling pathways, and heat-shock proteins 70 and 90 (Figure 5A). Our findings are in line with Skynner et al. [29] who also showed a significant alteration in the morphology of the hippocampus and cerebral cortex in relation to cell morphology, and cellular assembly and organization. The decrease in brain weight seen in our study was similar to the findings of Devries and Bentson et al. [30,31], who also showed an increase in brain atrophy in chronically corticosteroid-treated patients. The main pathway that we could identify in the rats’ brain tissue was related to Akt signaling with decreased abundance of microtubule-associated tau protein (Mapt) [32]. The cytoskeletal Tau protein is a crucial regulator of neuronal malfunction found in stress-driven hippocampal pathology. Its decrease or absence blocks the stress-evoked hippocampal synaptic signaling and morpho-functional damages related to both neuronal structure and connectivity as well as subsequent behavioral deficits [33]. The decrease in the levels of Mapt was also shown by Haynes et al. [34], who showed that systemic administration of dexamethasone in rats caused a dose–dependent decrease in immunoreactivity to Mapt and activation of the microglia. The proteomic changes in the brain were confirmed by the serum metabolomic changes identified in our previous study that showed an increase in levels of kynurenine, 3-hydroxy-kenurenine, tryptophan, and secondarily serotonin that inhibited extracellular dopamine and glutamate release [35].

#### 3.1.2. Proteins Related to Decreased Heart Size after Dex Treatment

Exogenous glucocorticoids such as Dex influence normal development and function of the heart and are known to have both positive and negative effects on it, although their direct role on signaling pathways remains unknown [36]. Prolonged Dex treatment leads to cardiac hypertrophy leading to the development of hypertension and insulin resistance. In their study, Ren et al. [37] found an increase in the hypertrophic markers that included an atrial natriuretic factor, β-myosin heavy chain and skeletal muscle α-actin. In growing animals, administration of Dex also causes decreased somatic growth and an increase in atherosclerotic events due to the presence of dyslipidemia [38]. The Dex-treated rats’ heart proteomes showed an increase in the levels of the enzyme hydroxysteroid 11-beta dehydrogenase 1(HSD11B1), which is known to augment GC action and increase the adverse effects of the drug, leading to increased insulin resistance, dyslipidemia, and hypertension [39]. The highest scoring network in the heart tissue between the Dex-treated and controlled rats related to pathways that were involved in cellular development, cellular function and maintenance, and connective tissue development and function. The central nodes were related to Akt and protein kinase C signaling pathways and also involved the heat-shock proteins 5 and 90 (Figure 5B). The heat-shock proteins functionally bind the GR to keep it in the normal inactive state and are released following Dex treatment for the translocation of the hormone-receptor complex into the nucleus for gene expression [23]. Involvement of the Akt signaling pathway in the heart tissue indicates the involvement of non-genomic actions of Dex in the development of side effects in the heart characterized by its reduced size compared to the controls. This can be related to the repressive effect of Dex on the proteins involved in the cell cycle [40] leading to suppression of the growth of cardiomyocytes [41] and a decrease in heart weight due to the inhibition of myocyte mitotic activity [30].

#### 3.1.3. Proteins Related to Muscle Atrophy after Dex Treatment 

Dexamethasone is a well-known inducer of muscle atrophy and myopathy [42] and is considered one of the causes for increased weight loss [43]. Prolonged use of the glucocorticoid is also known to have a catabolic and atrophic effect on skeletal muscle through increased gluconeogenesis [44], interfering with insulin-like growth factor-1 signaling [45] and influencing Akt1 signaling [46], resulting in increased degradation of muscle proteins. We found that the proteins identified in the muscle between the Dex-treated rats’ and controls’ datasets generated a network pathway which related to nucleic acid metabolism, small molecule biochemistry and vitamins, and mineral metabolism. It was interesting to note that the central nodes of the network were involved in the regulation of Akt, ERK, Jnk, AMPK, and the insulin signaling pathways (Figure 5C). Involvement of these pathways shows that Dex influences glucose metabolism, causes decreased protein synthesis and increased proteolysis, and causes morphological and functional damage to the muscle precursor cells such as the myoblast and also induces apoptosis. Involvement of AMPK signaling pathway, which acts on glucose utilization by increasing the expression of Glut4, and ERK signaling pathway also points to the important role of Dex in the regulation of insulin signals in muscle cells that have developed endoplasmic reticulum stress. These perturbations all relate to the development of insulin resistance myopathy observed post-Dex treatment [47]. Our findings are further confirmed with the distinct amino acid profile determined through our previous metabolomics study that showed decreased protein synthesis and an increased proteolysis characterized by an increase in the levels of amino acid glutamine by promoting protein catabolism.

#### 3.1.4. Proteins Altered in the Liver After Dex Treatment

In the liver, treatment with Dex changes the expression of the different hepatic enzymes, and increases the activity of key enzymes of the lipid metabolism, gluconeogenic pathway, the amino acids metabolism, the urea cycle [48], and the cytochrome p450 system which regulate many xenobiotic-metabolizing enzymes. In addition to these molecular effects, it also leads to morphological changes such as hepatomegaly and fatty liver [22,49] due to the hepatocyte hypertrophy from increased glycogen storage or fat accumulation [50]. A recent study by Ayyar et al. [51] studied the proteome-wide effects of methylprednisolone and its responses in the rat liver by using a functional pharmacoproteomic approach. They identified differential regulation of proteins related to various aspects of energy metabolism, amino acid metabolism, carbohydrate metabolism, lipid/fatty acid metabolism, and the Krebs cycle. The liver protein dataset identified in our study also pointed to a similar increase in the proteins involved in lipid metabolism, and gluconeogenic and urea cycle pathways. The highest scoring network pathway, identified using the IPA, related to lipid metabolism, small molecular biochemistry, and the nucleic acid metabolism pathway. The central nodes of the network were found to focus around an increase in the cytochrome P450 reductase system and involved the Akt and PI3 kinase signaling pathways (Figure 5D). Involvement of these signaling pathways has been implicated in a number of physiological cellular responses including survival, proliferation, protein synthesis, migration, vesicular trafficking, and increased mitochondrial respiration, as well as in pathogenesis of multiple diseases ranging from chronic inflammation to cancer [52]. These findings are also in line with the metabolomics study that found an increase in the levels of the acylcarnitines indicating an increased lipolytic state and an increase in gluconeogenic amino acids indicating an increase in gluconeogenesis [53].

#### 3.1.5. Proteins Altered in the Kidney After Dex Treatment

Dexamethasone is known to exert a direct action on the kidney on the renal tubular cells as well as the podocytes. It increases the renal vasodilatory effect, diuresis, the glomerular filtration rate, and the renal plasma flow [54]. The actions of Dex on the kidney are attributed to its inhibition of NFκB, which abrogates the inflammatory effects on both proteome and metabolome [55], and by increasing levels of angiotensin II and fibronectin which have been suggested to contribute to renal tubular fibrosis [56]. The protein dataset identified from the kidney in our study, when entered into the pathway analysis, identified with the highest score the network related to drug metabolism, organismal injury and abnormalities, and renal damage (Figure 5E). The central nodes of the network identified the involvement of insulin, ERK ½, p38 MAPK, Jnk, and NFκB signaling pathways highlighting the involvement and cross-talk among these related nodes. The coordinated stimulation of NFkB and Jnk cascades by inflammatory cytokines demonstrated by an increase in interleukins, IL 1 and IL18, in our dataset points towards alterations in proteins involved in glucose metabolism, apoptosis, and inflammation in the kidney, post-Dex treatment [57]. Involvement of these signaling pathways indicates an increase in Dex-mediated genomic regulation within the rat kidney that may lead to the development of side effects [3]. 

We found that among all five organs, the single signaling pathway that emerged as the point of commonality for the action of dexamethasone involved the Akt signaling pathway. This indicates that, more than the genomic actions, the development of side effects is due to the non-genomic actions of Dex mediated vis-a-vis the cytosolic GR. The GR, thus, mediates both the well-known genomic actions of the corticosteroid and is also involved in its much rapid non-genomic effects due the to complex interactions with various signaling processes [3].

#### 3.1.6. Proteins Altered in All Organs Treated with Dex

The proteins identified in the different organs between the Dex-treated and control rats were grouped together, and an additional bioinformatics analysis was carried out to get an overall view of the general effect of prolonged Dex treatment. The network analysis of all the differentially expressed proteins identified the network related to endocrine system development and function and drug metabolism and biochemistry, with a score of 83 (Figure S1). The highlighting of this pathway is in line with the different adverse effects that were previously seen with prolonged Dex use on the neuroendocrine, cardiovascular, and renal systems [58].

### 3.2. Comparison Amongst the Morhologically Altered Organs after Dex Treatment

The datasets were explored to identify if the proteomes of the tissues under study had proteins in common among them. We found that the proteomic profiles of these tissues after prolonged Dex treatment differed substantially from one another (Figure 2). Among all organs, the highest number of proteins in common was found between the heart and brain (*n* = 49), followed by the heart and liver (*n* = 39), and lastly the liver and brain (*n* = 30). On further probing, we found that eight proteins were in common between these three tissues (Figure 3 and Table 1). The other organs showed less similarity with regards to the differentially abundant proteins (Figure 3). What was intriguing was that we found no proteins in common between the muscle and the kidney datasets, which goes to show that the mechanism of action of Dex in these two organs is different from the others. The top canonical pathways affected by prolonged Dex treatment were different for all the organs while those pathways found in common between the organs had varied involvement. The liver, kidney, and heart protein datasets showed the LPS/IL-1-mediated inhibition of RXR function (10.4%, 3.7%, 6.6%, respectively) in common, the kidney and heart datasets had glutathione-mediated detoxification (10% and 25%, respectively), while the muscle and brain showed the involvement of glutathione redox reaction (8.7% and 22.7%, respectively). These findings demonstrate the differences in the degrees of involvement of the metabolic pathways in different organs by Dex probably due to the differences in the glucocorticoid receptor isoforms and their activity. 

A direct comparison of our results to other proteomic studies was difficult due to the differences in experimental techniques, duration, and type of treatments used, although similar results have been seen [18,29,51,59]. Our present study has, for the first time, demonstrated the effects of the treatment with Dex simultaneously on the major organ targets. Through this study, we have carried out a proteome mapping of each organ and demonstrated the immense complexity and differential actions of Dex within these different tissues, in essence, evaluating the drug’s effects. 

## 4. Materials and Methods

### 4.1. Ethical Considerations

Prior to implementation, all procedures and protocols for the animal studies were reviewed and approved by the Animal Care and Use Committee (ACUC) at King Faisal Specialist Hospital and Research Center (KFSHRC) (approval number RAC2150016). The animal model details were published elsewhere [10], where Sprague–Dawley (SD) rats were housed in the animal facility of the Department of Comparative Medicine at KFSHRC (Riyadh, Saudi Arabia). 

### 4.2. Experimental Design

A standard protocol of housing the study’s male SD rats was adopted, where the age of the rats ranged from 6 to 8 weeks (weight: 200–250 g). These rats were housed at the Department of Comparative Medicine under standard environmental conditions: temperature (20–24 °C), humidity (45–50%), and 12 h/12 h light/dark cycle with free access to food and water. Rats were randomly separated into two groups; Dex (*n* = 10) and control (*n* = 10) group. The Dex group was injected intramuscularly with 2.5 mg/kg twice a week for 14 weeks with Dex while the control with normal saline. This method was developed earlier by Li et al. [60] and Huang et al. [61] who considered giving equivalent long-term treatment of the drug for a chronic disease to develop the serious side effects of the drug. The clinical phenotype, anesthesia protocol, and the routine blood work analysis were monitored during this study and detailed previously [10]. After 14 weeks post-treatment, the animals were sacrificed (*n* = 5/group) and their major organs (brain, heart, muscle, liver, and kidney) collected, snap-frozen in liquid nitrogen, and then stored at −80 °C for further proteomics analysis. The other 5 animals included in the study were used for radiological studies as reported elsewhere [10].

### 4.3. Chemicals and Reagents

The dexamethasone phosphate drug, analytical solvents, and other standard chemicals for proteomics were obtained from Sigma–Aldrich (St. Louis, Missouri, USA). The reagents for routine chemistry analyses were purchased from Roche (Kaiseraugst, Switzerland).

### 4.4. Proteomics

#### 4.4.1. Sample Preparation for Label-Free Protein In-Solution Digestion

Homogenized tissue lysates were prepared from rat brain, heart, muscle, liver, and kidney tissues and were subjected to proteomic analysis (Figure 1). For each sample, 100 µg total protein extract was subjected to in-solution tryptic digestion, as previously described [62] Briefly, proteins were denatured in 0.1% RapiGest SF (Waters, Manchester, UK) at 80 °C for 15 minutes, reduced in 10 mM Dithiothreitol (DTT) at 60 °C for 30 min, and alkylated in 10 mM Iodoacetamide (IAA) (1.0 µL IAA/10 µL) in the dark for 40 min at room temperature. Samples were trypsin-digested overnight at 37 °C and the RapiGest reaction quenched with 12 M HCl. All samples were diluted with aqueous 0.1% formic acid before to achieve concentrations of 1 μg/μL before LC-MS/MS analysis.

#### 4.4.2. Protein Identification by LC-MSE SynaptG2 Platform

We undertook label-free quantitative expression profiling using 1-dimensional nanoACQUITY liquid chromatography combined with tandem mass spectrometry on a Synapt G2 HDMS instrument (Waters Scientific, Berkshire, UK). The electrospray ionization (ESI) mass spectrometry analyses were optimized and all acquisitions were carried out on a Trizaic Nano source (Waters) ionization in the positive ion mode nanoESI as previously described [62]. Briefly, the Mass Lynx IntelliStart was used to adjust for optimal instrument parameters including the following: detectors were set-up using 2 ng/μL leucine enkephalin (556.277 Da) and lock mass set-up and mass (*m/z*) calibration were achieved on a separate infusion line of 500 fmol (Glu) 1-fibrinopeptide B (GluFib, 785.8426 Da, capillary voltage 3 kV, sample cone 50 V, and extraction cone 5 V). Other parameters included source temperature 85 °C, cone gas 8 L/h, nano flow gas 0.5 bar, and purge gas 600 L/h.

Equal amounts of protein digest (3 μg) was loaded on a column (AcquityTrizaic Nano tile HSS T3 1.8 μm, 85 μm × 100 μm, Waters) and samples were processed using the Acquity sample manager with mobile phase comprising of A1 (99% water/1% acetonitrile/0.1% formic acid) and B1 (100% acetonitrile + 0.1% formic acid) with sample flow rate of 1 μL/min. Data independent acquisition (MS^E^)/iron mobility separation analyses were performed and data were acquired over a range of *m/z* 50–2000 Da with a scan time of 0.9 s and incremental transfer collision energy 20–50 V with a total acquisition time of 120 min. Data were acquired using the Mass Lynx program (version. 4.1, SCN833; Waters) operated in resolution and positive polarity modes and each sample was analyzed in triplicate runs (as a measure of reproducibility) and all samples were analyzed in the same batch run. 

All automated data processing and database searching was done using Progenesis QI for proteomics (ProgenesisQIfp version 2.0.5387, Nonlinear Dynamics/Waters, London, UK). The generated peptide masses were searched against the non-redundant species-specific protein sequence in the Uniprot database [63].

### 4.5. Data Analysis and Informatics

Progenesis QI for proteomics (ProgenesisQIfp version 2.0.5387) was used to process all Mass Lynx generated raw data and database search for protein identification. Each run in the experiment was represented as an ion intensity map, including the m/z and retention time, aligned and propagated for peak picking and peptide abundance measurements. The generated list of peptide ions for identification was subjected to a static database for searching using a search engine in Progenesis and the list of identified protein datasets were reviewed using multivariate statistical analyses.

A rat-specific database containing thousands of reviewed entries from Uniprot was created prior to searching as previously described [62,64]. The following criteria were used for the search: one missed cleavage, maximum protein mass 1000 kDa, trypsin, carbamidomethyl C fixed, and oxidation M variable modifications. Normalized label-free quantification was achieved and subjected to statistical analyses to plot principal component analysis (PCA) against data split into multiple groups. The data were filtered to show only statistically (ANOVA) significantly altered proteins (*p* ≤ 0.05) with ≥3 peptides identified, and a fold change of more than 1.5 was considered as the significant minimum threshold. Additionally “Hi3” absolute quantification was performed using alcohol dehydrogenase (ADH P00330) as an internal standard to give an absolute amount of each identified protein (Waters) as previously described [65]. These features are available as incorporated into ProgenesisQIfp (Nonlinear Dynamics/Waters, London, UK).

The analysis features available in ProgenesisQIfp (Nonlinear Dynamics/Waters) allows data to be filtered to show only statistically (ANOVA) significantly altered proteins as well as fold change in the levels of protein expression among sample pairs being compared. In this instance, we chose the level of significance at the 95% confidence interval, i.e., *p* ≤ 0.05, while we chose a fold change of more than 1.5 as the significant minimum threshold. Each sample was run in triplicates and the program took the average of the three runs and used the value in the expression ration of one protein among samples being compared. 

The proteomics expression profile was analyzed using MetaboAnalyst version 3.0 (McGill University, Montreal, Canada) [66]. The protein expression raw data among the study groups for each organ were uploaded to the software. The datasets were normalized to their sample total median for the specific organ set to ensure all samples were normally distributed. To visualize the proteomics differences among the study groups and make individual features more comparable, data were log-transformed and Pareto scaled, respectively. As the data were Gaussian distributed, the unpaired two-tailed Student’s *t*-test was used for binary comparison between the different study groups. Proteins were considered significant if they had an FDR-corrected *p*-value < 0.05 with 1.5 cut of fold change and visualized in a volcano plot.

### 4.6. Bioinformatics Analysis of Proteins in the Tissues and Network Pathway Analysis:

To understand the role of the identified proteins and interrogate the intracellular signaling pathways affected by alterations in these cellular proteins, gene ontology annotations were ascribed and network pathway analysis for the respective protein expression datasets for each organ were carried out. The quantitative protein data were imported into IPA software (Ingenuity^®^ Systems, [67]) for identification of the protein–protein interactions. This software also helps to determine the functions and pathways most strongly associated with a protein list by overlaying experimental expression data on networks constructed from published interactions. The identified networks help to infer the identity of upstream regulatory molecules and the associated mechanisms to provide biological insight to the observed experimental expression changes seen by the proteomics experiment. A score greater than three (*p* ≤ 0.001) indicates a greater than 99.9% confidence that a protein network was not generated by chance alone [68]. The score does not indicate the quality or significance of the network, it simply calculates the approximate “fit” between the network and the focus protein from the input dataset. The networks generated by this approach are preferentially enriched for proteins with the most extensive and specific interactions. The interacting proteins are represented as nodes, and the biological relationship between two nodes is represented as a line. Although powerful, the biological significance of the network(s) identified by this approach must ultimately be verified by experiment.

## 5. Conclusions

Our study is the first to use an untargeted LC-MSMS mass spectrometric approach to carry out a simultaneous comprehensive organ-specific proteomic profiling in SD rats after prolonged Dex treatment. The proteome of the five different organs was identified, explored, and systematically mapped to diverse molecular network pathways that were functionally influenced by the treatment. Identification of these pathways will help in better understanding the protein–protein interactions, their dynamics, and the proteomic and molecular signatures of the drug’s effects and side effects within them. These proteins can be developed into markers with potential use in monitoring for adverse effects and developing strategies towards preventing them.

## Figures and Tables

**Table 1:** This table shows the identity (gene name) of the proteins found to be overlapping between the different organs. The proteins found in common among the different organs are marked as: + common in heart, brain, liver; ++ common in heart, brain, kidney; ‡ common in kidney, heart, liver; ‡‡ liver, muscle, brain; † common in brain, heart, muscle; and †† common in brain, kidney, liver.

	Heart			Muscle	Liver			Kidney
**Brain**	*Acot1^+^* *ACOT7* *Aldh1a7* *AP2A2* *AP2M1* *ATP2B2* *ATP5PF^++^* *ATP6V0A1* *CEP83* *CES1* *CHDH^†^* *CLTRN* *CPS1^+^* *CSNK2A1*	*Cyp2d1/Cyp2d5^+^* *DBI* *ECHDC3* *EIF2D* *EPHX1* *GSTZ1^+^* *HEATR6* *IDH1* *MPC2^++^* *Mug1^+^* *MYL2^+^* *NENF* *PAFAH1B2* *CTSC*	*PDIA6* *PNP* *PYGL^+^* *RAB7A* *RALA* *RNLS* *RPS2* *RPS16^+^* *STAMBP* *STIP1* *STX1A* *SYN2* *Wasl*	*ATP5F1B* *CHDH^†^* *HPX* *MGST1* *PRDX6* *RIPOR2^‡‡^*	*Acot1* *AKR1A1* *AKR1B1* *BCKDHA* *CA1* *COX5B* *CPS1* *Cyp2d1/Cyp2d5* *GAPDH^††^* *GCSH* *GDI1* *GSTZ1* *HADH* *HMGCS1* *KHK*		*LDHB* *LRRC8* *CLYPLA1* *Mug1 * *MYH6* *MYL2* *PHYHIPL* *PYGL* *RIPOR2^‡‡^* *RPS16* *SLC25A4* *SLC25A5* *SUOX* *Tpm2* *VPS45*	*ATP5PF^++^* *CAPZB* *CORO6* *COTL1* *CSRP1* *GAPDH^††^* *GRN* *MCCC1* *MPC2^++^* *MYH6* *NCALD* *RAB3A* *RAB3C* *SCARB1* *UCMA*
**Heart**				*CHDH* *EZR* *NDUFA10* *RPSA*	*Acot1^+^* *ACOX1* *Akr1c14* *ALDOB* *AMACR* *ASS1* *ATP5PO* *BAAT* *BHMT2* *BIN1* *Ces1e* *CPS1^+^*	*CSRP3* *Cyp2d1/Cyp2d5^+^* *EEF1A2* *FABP1* *FABP3* *FBP1* *FHL2* *Gnmt* *Gsta1* *GSTA3* *GSTM2* *GSTZ1^+^*	*HMGCS2* *HSPA5* *MAT1A* *Mug1^+^* *MYL2^+^* *NDUFA9* *PYGL^+^* *RPS16^+^* *Sult1a1* *TKFC* *UGT2B15* *UPB1*	*ALDH1B1* *ATP5PF* *BGN* *CDKL3* *FABP1^‡^* *GSTM1* *GSTM2^‡^* *Hsd3b4 * *MPC2* *THNSL2* *TXNRD1* *Vamp1*
**Liver**				*ABHD17B* *ACD* *ACTN1* *CS* *IDH3A* *KRT24* *PC* *RIPOR2^‡‡^*				*AHSG* *FABP1^‡^* *GSTM2^‡^* *PDHB* *PKM* *Tpm3* *GAPDHS* *MYH6*

## Supplementary Materials

Supplementary materials can be found at https://www.mdpi.com/1422-0067/20/13/3122/s1. **Table S1.** List of differentially expressed significant proteins, with the fold change and *p*-values, identified in different organs between Dex-treated versus control SD rats using a label-free quantitative LC-MS/MS approach. **Table S2**. Table showing the abbreviations (gene name) and full name for all proteins mentioned in Table 1. **Table S3**: Table showing the top three molecular and cellular functional categories significantly altered in each organ after long-term Dex treatment with their *p*-value range, number of molecules involved, and the gene names of the involved proteins. **Table S4:** Table showing the IPA identified canonical pathways altered by Dex treatment. **Figure S1**. Network analysis of all the differentially expressed proteins identified in the different organs between the Dex-treated and control rats. The network identifies an integrated pathway that demonstrates the overall effect of prolonged dexamethasone in the treatment versus the control rats with the network related to drug metabolism, endocrine system development and function, and biochemistry with the score of 83.
