# Supplementary material for: Proteomic Analysis of Morphologically Changed Tissues after Prolonged Dexamethasone Treatment
Source: Int J Mol Sci. 2019 Jun 26;20(13):3122. doi: 10.3390/ijms20133122 (PMC6650964; doi:10.3390/ijms20133122)
Supplement: Supplementary file 1 [file ijms-20-03122-s001.zip › TableS1.pdf]

Table S1					
	**Accession No**	**Description**	**Expr p-value**	**Maximum Fold Change Value Dex/Control**	**Fold Change Dex/Control**
Brain					
1	Q63081	Protein disulfide-isomerase A6	2.76E-06	1.97	Down
2	Q920J3	Coronin-6	6.03E-06	3.65	Down
3	Q4FZY0	EF-hand domain-containing protein D2	6.93E-06	Infinity	Down
4	Q5XIN6	Mitochondrial proton/calcium exchanger protein	8.43E-06	5.26	Down
5	Q5U2W9	Renalase	1.47E-05	30.80	Down
6	Q63345	Myelin-oligodendrocyte glycoprotein	2.87E-05	2.14	Down
7	P63012	Ras-related protein Rab-3A	3.40E-05	1.71	Down
8	Q02563	Synaptic vesicle glycoprotein 2A	3.96E-05	2.26	Down
9	Q6P6R2	Dihydrolipoyl dehydrogenase, mitochondrial	5.21E-05	2.14	Down
10	P70619	Glutathione reductase (Fragment)	6.46E-05	2.09	Down
11	P97943	Scavenger receptor class B member 1	7.56E-05	4.08	Down
12	P48679	Prelamin-A/C	2.10E-04	1.81	Down
13	O08700	Vacuolar protein sorting-associated protein 45	2.14E-04	2.89	Down
14	Q5EAJ6	Inhibitor of nuclear factor kappa-B kinase-interacting protein	1.86E-04	2.39	Down
15	Q6P6V0	Glucose-6-phosphate isomerase	1.48E-04	2.11	Down
16	P56741	Myosin-binding protein C, cardiac-type	9.35E-05	1.70	Down
17	P12839	Neurofilament medium polypeptide	2.52E-04	1.66	Down
18	Q62636	Ras-related protein Rap-1b	2.77E-04	2.10	Down
19	P84092	AP-2 complex subunit mu	3.10E-04	2.06	Down
20	P07687	Epoxide hydrolase 1	3.13E-04	2.06	Down
21	Q8VD83	Ribonuclease pancreatic	3.27E-04	2.44	Down
22	G3V7W1	Programmed cell death protein 6	3.42E-04	1.86	Down
23	Q498T9	Volume-regulated anion channel subunit LRRC8C	3.42E-04	2.18	Down
24	O35763	Moesin	4.25E-04	1.84	Down
25	Q4KM74	Vesicle-trafficking protein SEC22b	4.70E-04	2.44	Down
26	Q05683	Glutamate decarboxylase 2	4.65E-04	2.72	Down
27	Q5EB92	WD repeat-containing protein 70	6.59E-04	3.82	Down
28	P80067	Dipeptidyl peptidase 1	6.93E-04	3.52	Down
29	P08503	Medium-chain specific acyl-CoA dehydrogenase, mitochondrial	7.06E-04	1.55	Down
30	P62824	Ras-related protein Rab-3C	7.67E-04	1.72	Down
31	O70199	UDP-glucose 6-dehydrogenase	8.22E-04	1.66	Down
32	P01026	Complement C3	8.24E-04	2.07	Down
33	P41562	Isocitrate dehydrogenase [NADP] cytoplasmic	8.51E-04	1.50	Down
34	P32851	Syntaxin-1A	1.19E-03	2.15	Down
35	P07314	Gamma-glutamyltranspeptidase 1	1.28E-03	3.07	Down
36	Q4KM49	Tyrosine--tRNA ligase, cytoplasmic	1.61E-03	1.61	Down
37	Q63413	Spliceosome RNA helicase Ddx39b	1.80E-03	1.92	Down
38	Q9WVK7	Hydroxyacyl-coenzyme A dehydrogenase, mitochondrial	2.01E-03	1.52	Down
39	Q62722	NGFI-A-binding protein 1	2.06E-03	1.69	Down
40	Q63530	Phosphotriesterase-related protein	2.10E-03	1.95	Down

41	P11960	2-oxoisovalerate dehydrogenase subunit alpha, mitochondrial (Fragment)	2.14E-03	3.86	Down
42	P80254	D-dopachrome decarboxylase	2.14E-03	7.88	Down
43	O35264	Platelet-activating factor acetylhydrolase IB subunit beta	2.41E-03	1.62	Down
44	Q6AY56	Tubulin alpha-8 chain	2.99E-03	2.43	Down
45	P02563	Myosin-6	3.13E-03	2.05	Down
46	Q5U316	Ras-related protein Rab-35	3.65E-03	2.66	Down
47	P67779	Prohibitin	3.75E-03	1.67	Down
48	O35854	Branched-chain-amino-acid aminotransferase, mitochondrial	3.89E-03	1.92	Down
49	Q5XIT9	Methylcrotonoyl-CoA carboxylase beta chain, mitochondrial	4.16E-03	1.71	Down
50	P63245	Receptor of activated protein C kinase 1	4.35E-03	2.00	Down
51	P25286	V-type proton ATPase 116 kDa subunit a isoform 1	4.45E-03	3.33	Down
52	P13601	Aldehyde dehydrogenase, cytosolic 1	4.90E-03	1.79	Down
53	P08011	Microsomal glutathione S-transferase 1	5.23E-03	2.43	Down
54	Q6AY09	Heterogeneous nuclear ribonucleoprotein H2	5.90E-03	3.87	Down
55	D3Z8N2	E3 ubiquitin-protein ligase RNF187	5.90E-03	1.60	Down
56	P20673	Argininosuccinate lyase	6.55E-03	1.77	Down
57	Q9WUW8	Sulfotransferase 1C2	6.60E-03	1.86	Down
58	P06866	Haptoglobin	6.77E-03	1.92	Down
59	P12939	Cytochrome P450 2D10	6.94E-03	5.10	Down
60	Q02974	Ketohexokinase	7.20E-03	4.81	Down
61	P85973	Purine nucleoside phosphorylase	7.28E-03	2.32	Down
62	D3ZGQ5	Serine/threonine-protein kinase Nek8	7.29E-03	4.98	Down
63	P20059	Hemopexin	8.00E-03	2.11	Down
64	Q7TP54	Rho family-interacting cell polarization regulator 2	8.63E-03	1.87	Down
65	Q62838	Muscle, skeletal receptor tyrosine protein kinase	8.76E-03	3.53	Down
66	Q5PPG7	Eukaryotic translation initiation factor 2D	1.09E-02	1.52	Down
67	P09811	Glycogen phosphorylase, liver form	9.52E-03	5.15	Down
68	O54975	Xaa-Pro aminopeptidase 1	1.01E-02	1.66	Down
69	P07379	Phosphoenolpyruvate carboxykinase, cytosolic [GTP]	1.06E-02	1.60	Down
70	P18484	AP-2 complex subunit alpha-2	1.11E-02	2.37	Down
71	P63331	Serine/threonine-protein phosphatase 2A catalytic subunit alpha isoform	1.17E-02	2.96	Down
72	B9TQX4	Unique cartilage matrix-associated protein	1.31E-02	10.03	Down
73	P05544	Serine protease inhibitor A3L	1.37E-02	1.74	Down
74	Q66HA6	ADP-ribosylation factor-like protein 8B	1.46E-02	1.96	Down
75	P18291	Granzyme B	1.49E-02	44.15	Down
76	Q6UPE0	Choline dehydrogenase, mitochondrial	1.91E-02	4.67	Down
77	P51635	Alcohol dehydrogenase [NADP(+)]	2.05E-02	1.76	Down
78	Q5XI32	F-actin-capping protein subunit beta	2.22E-02	1.79	Down
79	O35814	Stress-induced-phosphoprotein 1	2.34E-02	1.61	Down
80	Q9ESG3	Collectrin	2.35E-02	1.62	Down
81	P54921	Alpha-soluble NSF attachment protein	2.46E-02	1.63	Down
82	P07943	Aldose reductase	2.74E-02	2.09	Down
83	P13471	40S ribosomal protein S14	2.91E-02	1.69	Down
84	Q68G31	Phenazine biosynthesis-like domain-containing protein	3.06E-02	1.87	Down

85	P50398	Rab GDP dissociation inhibitor alpha	3.17E-02	1.69	Down
86	O08816	Neural Wiskott-Aldrich syndrome protein	3.21E-02	2.31	Down
87	Q4V8I7	Volume-regulated anion channel subunit LRRC8A	3.37E-02	1.61	Down
88	Q6AYN4	Phytanoyl-CoA hydroxylase-interacting protein-like	3.42E-02	1.51	Down
89	P19332	Microtubule-associated protein tau	3.47E-02	1.66	Down
90	Q91ZW1	Transcription factor A, mitochondrial	3.49E-02	1.88	Down
91	Q5U1Y1	Ras-related protein Rab-34	3.52E-02	2.06	Down
92	P57113	Maleylacetoacetate isomerase	3.68E-02	2.92	Down
93	P17425	Hydroxymethylglutaryl-CoA synthase, cytoplasmic	3.98E-02	1.92	Down
94	P07756	Carbamoyl-phosphate synthase [ammonia], mitochondrial	4.12E-02	1.72	Down
95	Q6IE52	Murinoglobulin-2	4.20E-02	1.81	Down
96	Q6AYS7	Aminoacylase-1A	4.21E-02	2.13	Down
97	Q5BJY9	Keratin, type I cytoskeletal 18	4.51E-02	1.69	Down
98	P08733	Myosin regulatory light chain 2, ventricular/cardiac muscle isoform	4.58E-02	1.51	Down
99	Q6MFY8	Tripartite motif-containing protein 40	4.59E-02	2.39	Down
100	Q63537	Synapsin-2	3.23E-07	2.78	UP
101	Q38RY9	Cytochrome c oxidase subunit 2	1.33E-06	Infinity	UP
102	P38718	Mitochondrial pyruvate carrier 2	2.46E-06	2452.79	UP
103	A9UMV8	Histone H2A.J	1.58E-05	4.76	UP
104	Q9ESV6	Glyceraldehyde-3-phosphate dehydrogenase, testis-specific	3.24E-05	1.94	UP
105	P04355	Metallothionein-2	8.70E-06	11152.34	UP
106	P23565	Alpha-internexin	3.09E-06	3.10	UP
107	A1EC95	HEAT repeat-containing protein 6	5.88E-05	2.98	UP
108	P0C0S7	Histone H2A.Z	6.01E-05	3.04	UP
109	Q75Q40	Mitochondrial import receptor subunit TOM40 homolog	8.01E-05	15.82	UP
110	P11517	Hemoglobin subunit beta-2	8.89E-05	1.69	UP
111	Q5FVI4	Cell cycle exit and neuronal differentiation protein 1	9.19E-05	1.66	UP
112	Q5PQN0	Neurocalcin-delta	3.81E-04	1.82	UP
113	Q6IUR5	Neudesin	9.67E-05	2.02	UP
114	P04631	Protein S100-B	1.27E-04	1.79	UP
115	P04797	Glyceraldehyde-3-phosphate dehydrogenase	1.95E-04	2.73	UP
116	Q63068	Phosphorylated adapter RNA export protein	1.70E-04	Infinity	UP
117	P09117	Fructose-bisphosphate aldolase C	3.39E-04	1.61	UP
118	P62804	Histone H4	4.34E-04	2.14	UP
120	O35331	Pyridoxal kinase	4.81E-04	2.14	UP
121	P63029	Translationally-controlled tumor protein	5.43E-04	1.73	UP
122	P35704	Peroxiredoxin-2	5.98E-04	1.77	UP
123	O88267	Acyl-coenzyme A thioesterase 1	2.17E-04	4.21	UP
124	B0BNA5	Coactosin-like protein	2.30E-04	2.64	UP
125	P09527	Ras-related protein Rab-7a	8.90E-04	3.61	UP
126	P24942	Excitatory amino acid transporter 1	9.46E-04	2.96	UP
127	A7VJC2	Heterogeneous nuclear ribonucleoproteins A2/B1	9.70E-04	1.62	UP
128	P19527	Neurofilament light polypeptide	1.32E-03	1.81	UP
129	O35796	Complement component 1 Q subcomponent-binding protein, mitochondrial	1.45E-03	1.64	UP

130	P45479	Palmitoyl-protein thioesterase 1	1.58E-03	4.24	UP
131	P00406	Cytochrome c oxidase subunit 2	1.59E-03	10.07	UP
132	Q5I0P2	Glycine cleavage system H protein, mitochondrial	2.52E-03	12.30	UP
133	P58775	Tropomyosin beta chain	2.54E-03	1.55	UP
134	Q6AXS3	Protein DEK	2.73E-03	Infinity	UP
135	Q00566	Methyl-CpG-binding protein 2	2.78E-03	6.43	UP
136	P06349	Histone H1t	2.78E-03	2.43	UP
137	P53767	Vascular endothelial growth factor receptor 1	3.28E-03	1.93	UP
138	Q9JJK1	Neuronal membrane glycoprotein M6-b	3.47E-03	1.68	UP
139	P05714	Ras-related protein Rab-4A	3.68E-03	4.59	UP
140	P04906	Glutathione S-transferase P	3.85E-03	2.13	UP
141	P51647	Retinal dehydrogenase 1	4.17E-03	1.69	UP
142	P16303	Carboxylesterase 1D	6.38E-03	2.09	UP
143	Q5I0C3	Methylcrotonoyl-CoA carboxylase subunit alpha, mitochondrial	7.05E-03	22.49	UP
144	Q8CFG5	Voltage-dependent calcium channel subunit alpha-2/delta-3	7.13E-03	16.10	UP
145	B0BNF1	Septin-8	9.03E-03	3.03	UP
146	Q6AXP3	Protein FAM227B	1.01E-02	3.42	UP
147	P49911	Acidic leucine-rich nuclear phosphoprotein 32 family member A	1.07E-02	2.10	UP
148	Q07116	Sulfite oxidase, mitochondrial	1.10E-02	1.76	UP
149	P42123	L-lactate dehydrogenase B chain	1.11E-02	1.77	UP
150	B2RYG6	Ubiquitin thioesterase OTUB1	1.30E-02	2.87	UP
151	P13638	Sodium/potassium-transporting ATPase subunit beta-2	1.40E-02	4.13	UP
152	O88377	Phosphatidylinositol 5-phosphate 4-kinase type-2 beta	1.46E-02	43.73	UP
153	Q0VGK3	Glycerate kinase	1.51E-02	Infinity	UP
154	P19139	Casein kinase II subunit alpha	1.52E-02	4.00	UP
155	Q05962	ADP/ATP translocase 1	1.74E-02	1.56	UP
156	B0BNN3	Carbonic anhydrase 1	1.86E-02	1.75	UP
157	O88780	Kallikrein-8	1.92E-02	2.16	UP
158	D3ZAF6	ATP synthase subunit f, mitochondrial	2.05E-02	1.64	UP
159	P23785	Granulins	2.06E-02	Infinity	UP
160	Q09073	ADP/ATP translocase 2	2.23E-02	1.60	UP
161	P27952	40S ribosomal protein S2	2.30E-02	2.91	UP
162	Q8R424	STAM-binding protein	2.33E-02	1.74	UP
164	P21571	ATP synthase-coupling factor 6, mitochondrial	2.42E-02	1.51	UP
165	P11030	Acyl-CoA-binding protein	2.62E-02	2.68	UP
166	Q66H89	Centrosomal protein of 83 kDa	2.75E-02	4.70	UP
167	Q9Z0J8	Neuronal growth regulator 1	2.77E-02	2.19	UP
168	P18421	Proteasome subunit beta type-1	2.87E-02	3.58	UP
169	P47875	Cysteine and glycine-rich protein 1	2.87E-02	3.18	UP
170	P60203	Myelin proteolipid protein	2.87E-02	2.82	UP
171	Q02874	Core histone macro-H2A.1	3.46E-02	2.35	UP
172	P62250	40S ribosomal protein S16	3.00E-02	5.60	UP
173	P11506	Plasma membrane calcium-transporting ATPase 2	3.05E-02	5.54	UP
174	P15865	Histone H1.4	3.18E-02	2.61	UP

175	Q6AYZ1	Tubulin alpha-1C chain	3.38E-02	2.37	UP
176	P46462	Transitional endoplasmic reticulum ATPase	3.52E-02	1.62	UP
177	P70470	Acyl-protein thioesterase 1	3.58E-02	2.35	UP
178	P10719	ATP synthase subunit beta, mitochondrial	3.61E-02	1.76	UP
179	Q4V8B3	Mediator of RNA polymerase II transcription subunit 24	3.91E-02	1.54	UP
180	P12075	Cytochrome c oxidase subunit 5B, mitochondrial	3.91E-02	3.48	UP
181	P52481	Adenylyl cyclase-associated protein 2	4.09E-02	3.98	UP
182	O35244	Peroxiredoxin-6	4.22E-02	1.52	UP
183	P49242	40S ribosomal protein S3a	4.31E-02	1.89	UP
184	Q8VD93	Ribonuclease pancreatic alpha-type OS=Rattus exulans PE=3 SV=1	4.35E-02	7.57	UP
185	P97697	Inositol monophosphatase 1	4.47E-02	2.33	UP
186	P63041	Complexin-1	4.51E-02	4.87	UP
187	P60868	40S ribosomal protein S20	4.70E-02	3.33	UP
188	Q3MIE0	Enoyl-CoA hydratase domain-containing protein 3, mitochondrial	4.79E-02	1.63	UP
189	Q68FU3	Electron transfer flavoprotein subunit beta	4.80E-02	2.29	UP
190	P63322	Ras-related protein Ral-A	4.82E-02	1.94	UP
191	Q64559	Cytosolic acyl coenzyme A thioester hydrolase	4.83E-02	1.58	UP
192	P13635	Ceruloplasmin	4.99E-02	2.19	UP

Heart					
1.	Q9ES21	SAC1 like phosphatidylinositide phosphatase	7.57E-06	6223.63	Down
2.	P10634	Cytochrome P450, family 2, subfamily d, polypeptide 26	1.26E-06	2715.07	Down
3.	Q9ES53	Ubiquitin recognition factor in ER associated degradation 1	6.67E-03	75.80	Down
4.	P85973	Purine nucleoside phosphorylase	5.57E-04	68.13	Down
5.	O89049	Thioredoxin reductase 1	9.42E-04	58.13	Down
6.	Q3MIE0	Enoyl-coa hydratase domain containing 3	2.86E-03	57.69	Down
7.	D3ZGS3	OCRL, inositol polyphosphate-5-phosphatase	2.66E-04	37.88	Down
8.	P07687	Epoxide hydrolase 1	1.07E-02	36.59	Down
9.	Q63276	Bile acid-coa:amino acid N-acyltransferase	1.69E-04	26.83	Down
10.	P23457	Aldo-keto reductase family 1, member C14	5.11E-03	22.56	Down
11.	P07150	Annexin A1	1.38E-03	20.30	Down
12.	P02692	Fatty acid binding protein 1	1.68E-02	19.83	Down
13.	B0K020	CDGSH iron sulfur domain 1	1.16E-02	16.47	Down
14.	Q6IUR5	Neudesin neurotrophic factor	4.02E-02	14.94	Down
15.	P17988	Sulfotransferase family 1A, phenol-preferring, member 1	4.82E-03	14.83	Down
16.	P63322	RAS like proto-oncogene A	6.62E-03	14.78	Down
17.	Q99P82	Claudin 11	5.42E-03	12.89	Down
18.	Q5PPJ9	SH3 domain-containing GRB2-like endophilin B2	2.04E-03	11.47	Down
19.	P06757	Alcohol dehydrogenase 1C (class I), gamma polypeptide	3.32E-05	10.95	Down
20.	P16303	Carboxylesterase 1	1.07E-03	10.94	Down
21.	P08683	Cytochrome P450 family 2 subfamily C member 9	4.98E-04	10.74	Down
22.	P62250	Ribosomal protein S16	1.09E-03	10.71	Down
23.	P14141	Carbonic anhydrase 3	2.71E-06	7.96	Down

24.	P07756	Carbamoyl-phosphate synthase 1	1.69E-05	7.06	Down
25.	P38718	Mitochondrial pyruvate carrier 2	1.73E-02	6.81	Down
26.	P10818	Cytochrome c oxidase subunit 6A1	1.50E-04	6.64	Down
27.	Q63544	Synuclein gamma	3.49E-02	6.14	Down
28.	P22791	3-hydroxy-3-methylglutaryl-coa synthase 2	1.34E-05	5.38	Down
29.	P57113	Glutathione S-transferase zeta 1	5.07E-04	5.16	Down
30.	Q5M7T9	Threonine synthase like 2	4.21E-03	4.31	Down
31.	P08542	UDP glucuronosyltransferase 2 family, polypeptide B15	3.13E-04	4.29	Down
32.	P00502	Glutathione S-transferase alpha 1	6.67E-03	4.06	Down
33.	P00884	Aldolase, fructose-bisphosphate B	2.02E-03	3.98	Down
34.	Q9JM01	Cyclin dependent kinase like 3	6.88E-06	3.89	Down
35.	Q63666	Vesicle-associated membrane protein 1	4.00E-04	3.60	Down
36.	P52759	Reactive intermediate imine deaminase A homolog	2.51E-04	3.58	Down
37.	P31977	Ezrin	7.41E-05	3.57	Down
38.	P04905	Glutathione S-transferase mu 5	9.49E-04	3.52	Down
39.	Q8VIF7	Selenium binding protein 1	1.83E-03	3.20	Down
40.	P09527	RAB7A, member RAS oncogene family	3.42E-02	3.15	Down
41.	B5DFI8	Ubiquitin conjugating enzyme E2 S	1.32E-02	3.04	Down
42.	P09034	Argininosuccinate synthase 1	1.02E-04	3.02	Down
43.	P51146	RAB4B, member RAS oncogene family	8.97E-03	3.02	Down
44.	P19112	Fructose-bisphosphatase 1	3.69E-04	3.00	Down
45.	P07824	Arginase 1	2.36E-04	2.99	Down
46.	Q812E9	Glycoprotein M6A	1.06E-03	2.96	Down
47.	D4AE41	RBMX like 1	7.19E-04	2.93	Down
48.	P48004	Proteasome subunit alpha 7	9.49E-03	2.92	Down
49.	Q63150	Dihydropyrimidinase	1.55E-03	2.89	Down
50.	Q5PQT3	Glycine-N-acyltransferase	1.65E-03	2.88	Down
51.	P85970	Actin related protein 2/3 complex subunit 2	6.11E-03	2.87	Down
52.	P62243	Ribosomal protein S8	7.91E-04	2.78	Down
53.	P18420	Proteasome subunit alpha 1	1.81E-02	2.68	Down
54.	P0DP29	Calmodulin 1	7.90E-04	2.68	Down
55.	P19488	UDP glucuronosyltransferase family 2 member B15	3.09E-03	2.68	Down
56.	Q03248	Beta-ureidopropionase 1	1.50E-02	2.65	Down
57.	P08010	Glutathione S-transferase mu 1	1.17E-03	2.64	Down
58.	P24470	Cytochrome P450, family 2, subfamily c, polypeptide 23	4.26E-02	2.58	Down
59.	P02401	Ribosomal protein, large P2	7.05E-03	2.54	Down
60.	P60522	GABA type A receptor associated protein like 2	4.89E-02	2.49	Down
61.	P20070	Cytochrome b5 reductase 3	4.18E-03	2.44	Down
62.	Q497B0	Nitrilase family member 2	1.01E-03	2.40	Down
63.	P61980	Heterogeneous nuclear ribonucleoprotein K	2.55E-03	2.40	Down
64.	P27867	Sorbitol dehydrogenase	6.38E-04	2.38	Down
65.	Q63108	Carboxylesterase 1E	2.64E-02	2.37	Down

66.	P00173	Cytochrome b5 type A	4.87E-04	2.36	Down
67.	Q63537	Synapsin II	7.92E-03	2.33	Down
68.	Q64559	Acyl-coa thioesterase 7	6.98E-03	2.32	Down
69.	P47853	Biglycan	3.07E-02	2.32	Down
70.	Q62625	Microtubule associated protein 1 light chain 3 beta	3.74E-03	2.32	Down
71.	P25286	Atpase H+ transporting V0 subunit a1	2.12E-04	2.25	Down
72.	Q6UPE0	Choline dehydrogenase	4.39E-04	2.23	Down
73.	P04904	Glutathione S-transferase alpha 1	4.18E-04	2.18	Down
74.	O09171	Betaine--homocysteine S-methyltransferase	6.48E-04	2.14	Down
75.	Q5PPG7	Eukaryotic translation initiation factor 2D	9.82E-03	2.14	Down
76.	P70473	Alpha-methylacyl-coa racemase	3.10E-03	2.14	Down
77.	Q4QRB4	Tubulin beta 3 class III	4.53E-02	2.08	Down
78.	P46418	Glutathione S-transferase alpha 3	3.21E-04	2.07	Down
79.	P04762	Catalase	1.98E-04	2.07	Down
80.	Q64638	UDP glucuronosyltransferase family 1 member A4	8.24E-05	2.05	Down
81.	P84092	Adaptor related protein complex 2 subunit mu 1	1.80E-04	2.02	Down
82.	P38918	Aldo-keto reductase family 7 member A3	3.73E-04	2.00	Down
83.	P18757	Cystathionine gamma-lyase	4.33E-03	1.99	Down
84.	Q64057	Aldehyde dehydrogenase 7 family member A1	3.90E-02	1.99	Down
85.	O35077	Glycerol-3-phosphate dehydrogenase 1	2.96E-04	1.98	Down
86.	Q7TP52	Carboxymethylenebutenolidase homolog	4.17E-03	1.88	Down
87.	P06761	Heat shock protein family A (Hsp70) member 5	5.31E-04	1.86	Down
88.	Q08163	Cyclase associated actin cytoskeleton regulatory protein 1	8.36E-03	1.82	Down
89.	Q9ESG3	Collectrin, amino acid transport regulator	4.41E-03	1.82	Down
90.	P07632	Superoxide dismutase 1	7.07E-04	1.80	Down
91.	P38983	Ribosomal protein SA	2.57E-02	1.79	Down
92.	Q561S0	NADH:ubiquinone oxidoreductase subunit A10	1.70E-02	1.78	Down
93.	Q5PPJ4	Deoxyhypusine hydroxylase	1.02E-02	1.75	Down
94.	Q4KLZ6	Triokinase and FMN cyclase	1.82E-02	1.74	Down
95.	Q68FT5	Betaine--homocysteine S-methyltransferase 2	6.27E-04	1.73	Down
96.	P11030	Diazepam binding inhibitor, acyl-coa binding protein	8.53E-03	1.72	Down
97.	P30904	Macrophage migration inhibitory factor	1.15E-02	1.71	Down
98.	P08009	Glutathione S-transferase mu 2	9.78E-03	1.68	Down
99.	P27364	Hydroxy-delta-5-steroid dehydrogenase, 3 beta- and steroid delta-isomerase 4	3.30E-02	1.66	Down
100.	P09811	Glycogen phosphorylase L	4.16E-02	1.65	Down
101.	Q62876	Synaptogyrin 1	1.30E-03	1.62	Down
102.	Q5BK61	Sorting nexin 20	2.35E-02	1.62	Down
103.	Q63081	Protein disulfide isomerase family A member 6	2.14E-02	1.61	Down
104.	Q9R1T5	Aspartoacylase	4.73E-03	1.61	Down
105.	P62278	Predicted gene 15483	3.93E-02	1.61	Down
106.	P19944	Ribosomal protein lateral stalk subunit P1	9.19E-03	1.61	Down
107.	Q68FR6	Eukaryotic translation elongation factor 1 gamma	4.07E-02	1.60	Down

108.	Q9WVK3	Peroxisomal trans-2-enoyl-coa reductase	3.99E-03	1.59	Down
109.	P80067	Cathepsin C	1.29E-03	1.58	Down
110.	P13437	Acetyl-coa acyltransferase 2	4.06E-03	1.58	Down
111.	O35303	Dynamin 1 like	2.72E-02	1.57	Down
112.	P13255	Glycine N-methyltransferase	7.24E-04	1.57	Down
113.	Q03336	Regucalcin	9.21E-04	1.56	Down
114.	P27952	Ribosomal protein S2	2.36E-02	1.54	Down
115.	Q6MG60	Dimethylarginine dimethylaminohydrolase 2	6.57E-03	1.50	Up
116.	Q6IE52	Murinoglobulin 1	4.13E-03	1.50	Up
117.	P18484	Adaptor related protein complex 2 subunit alpha 2	1.76E-03	1.51	Up
118.	P63086	Mitogen-activated protein kinase 1	3.77E-05	1.51	Up
119.	Q8VHV7	Heterogeneous nuclear ribonucleoprotein H1	1.17E-02	1.51	Up
120.	Q62812	Myosin heavy chain 9	7.47E-03	1.51	Up
121.	P16232	Hydroxysteroid 11-beta dehydrogenase 1	8.81E-03	1.52	Up
122.	P19139	Casein kinase 2 alpha 1	3.66E-02	1.52	Up
123.	P07483	Fatty acid binding protein 3	2.87E-02	1.52	Up
124.	P11442	Clathrin heavy chain	1.07E-04	1.53	Up
125.	Q06647	ATP synthase peripheral stalk subunit OSCP	1.87E-02	1.53	Up
126.	Q6PCU2	Atpase H+ transporting V1 subunit E1	1.01E-03	1.53	Up
127.	P12939	Cytochrome P450, family 2, subfamily d, polypeptide 1	1.32E-04	1.53	Up
128.	P62632	Eukaryotic translation elongation factor 1 alpha 2	1.16E-03	1.54	Up
129.	Q8R424	STAM binding protein	1.71E-02	1.54	Up
130.	P07872	Acyl-coa oxidase 1	2.13E-03	1.55	Up
131.	P09605	Creatine kinase, mitochondrial 2	1.79E-02	1.55	Up
132.	O35264	Platelet activating factor acetylhydrolase 1b catalytic subunit 2	1.24E-02	1.56	Up
133.	P63088	Protein phosphatase 1 catalytic subunit gamma	7.54E-04	1.56	Up
134.	P24053	Neuromedin B receptor	8.94E-03	1.56	Up
135.	Q9Z2M4	2,4-dienoyl-coa reductase 2	4.68E-02	1.57	Up
136.	O35814	Stress induced phosphoprotein 1	1.09E-03	1.57	Up
137.	Q53B90	RAB43, member RAS oncogene family	2.84E-02	1.58	Up
138.	Q9WTT6	Guanine deaminase	6.99E-03	1.60	Up
139.	Q920A6	Serine carboxypeptidase 1	1.75E-02	1.60	Up
140.	Q68FP1	Gelsolin	1.24E-02	1.61	Up
141.	P04256	Heterogeneous nuclear ribonucleoprotein A1	5.75E-03	1.61	Up
142.	O88267	Acyl-coa thioesterase 1	2.61E-03	1.61	Up
143.	P29147	3-hydroxybutyrate dehydrogenase 1	4.05E-03	1.62	Up
144.	Q66HF8	Aldehyde dehydrogenase 1 family member B1	7.02E-03	1.62	Up
145.	P50463	Cysteine and glycine rich protein 3	3.23E-03	1.62	Up
146.	O35095	Neurochondrin	1.81E-03	1.63	Up
147.	D4A1J4	3-hydroxybutyrate dehydrogenase 2	6.73E-04	1.67	Up
148.	P06238	Alpha-2-macroglobulin	4.84E-04	1.67	Up
149.	P70536	Oxytocin receptor	2.09E-04	1.68	Up

150.	Q4KM73	Cytidine/uridine monophosphate kinase 1	7.48E-05	1.69	Up
151.	Q5U3Z6	Deuterosome assembly protein 1	1.65E-03	1.69	Up
152.	P07722	Myelin associated glycoprotein	1.23E-02	1.70	Up
153.	P24464	Cytochrome P450 family 4 subfamily A member 22	1.08E-03	1.70	Up
154.	P62142	Protein phosphatase 1 catalytic subunit beta	4.29E-03	1.71	Up
155.	Q5BJL5	Strawberry notch homolog 1	2.18E-03	1.71	Up
156.	P13601	Aldehyde dehydrogenase family 1, subfamily A7	2.14E-04	1.72	Up
157.	P32851	Syntaxin 1A	7.88E-04	1.76	Up
158.	P09951	Synapsin I	4.84E-03	1.77	Up
159.	Q1WIM3	Cell adhesion molecule 3	4.81E-03	1.78	Up
160.	A1EC95	HEAT repeat containing 6	2.42E-05	1.78	Up
161.	P35171	Cytochrome c oxidase subunit viia polypeptide 2-like 2	5.53E-03	1.80	Up
162.	Q6XQN1	Nicotinate phosphoribosyltransferase	2.21E-02	1.80	Up
163.	P41562	Isocitrate dehydrogenase (NADP(+)) 1, cytosolic	1.52E-04	1.83	Up
164.	P01256	Calcitonin related polypeptide beta	2.09E-02	1.84	Up
165.	Q5U2W9	Renalase, FAD dependent amine oxidase	3.27E-03	1.88	Up
166.	O35115	Four and a half LIM domains 2	1.15E-03	1.89	Up
167.	O08838	Amphiphysin	5.52E-03	1.90	Up
168.	Q5XIU9	Progesterone receptor membrane component 2	1.31E-03	1.90	Up
169.	Q499N5	Acyl-coa synthetase family member 2	8.21E-04	1.90	Up
170.	Q4V7A0	WD repeat domain 61	7.73E-04	1.90	Up
171.	P08733	Myosin light chain 2	6.87E-03	1.90	Up
172.	P52590	Nucleoporin 107	1.19E-04	1.90	Up
173.	P10687	Phospholipase C beta 1	7.60E-04	1.91	Up
174.	Q63449	Progesterone receptor	4.92E-03	2.00	Up
175.	O08816	Wiskott-Aldrich syndrome-like (human)	3.85E-04	2.07	Up
176.	P55146	TYRO3 protein tyrosine kinase	1.11E-04	2.10	Up
177.	P21571	ATP synthase peripheral stalk subunit F6	4.25E-04	2.12	Up
178.	O88797	DAB2, clathrin adaptor protein	3.84E-04	2.15	Up
179.	P61751	ADP ribosylation factor 4	1.78E-04	2.16	Up
180.	Q63120	ATP binding cassette subfamily C member 2	6.08E-05	2.20	Up
181.	P11506	Atpase plasma membrane Ca2+ transporting 2	3.37E-02	2.22	Up
182.	Q66H89	Centrosomal protein 83	1.17E-02	2.26	Up
183.	Q6P9T8	Tubulin beta 4B class ivb	1.21E-03	2.28	Up
184.	Q5BK63	NADH:ubiquinone oxidoreductase subunit A9	4.72E-02	2.28	Up
185.	O08839	Bridging integrator 1	8.86E-03	2.30	Up
186.	P62944	Adaptor related protein complex 2 subunit beta 1	1.75E-02	2.38	Up
187.	P38652	Phosphoglucomutase 1	2.11E-02	2.61	Up
188.	P13444	Methionine adenosyltransferase 1A	8.14E-03	3.27	Up
189.	P84087	Complexin 2	3.73E-03	3.37	Up
190.	P35281	RAB10, member RAS oncogene family	1.93E-02	4.45	Up
191.	P35434	ATP synthase F1 subunit delta	3.29E-02	4.78	Up

192.	P06399	Fibrinogen alpha chain	2.17E-03	11.22	Up
193.	Q63228	Glia maturation factor beta	5.18E-05	500.00	Up

MUSCLE					
1	O09028	Gamma-aminobutyric acid receptor subunit pi	1.60E-04	Infinity	Down
2	P04182	Ornithine aminotransferase, mitochondrial	5.09E-03	3.07	Down
3	Q63518	Myosin-binding protein C, slow-type (Fragment)	6.82E-03	2.77	Down
4	P27605	Hypoxanthine-guanine phosphoribosyltransferase	1.12E-02	Infinity	Down
5	Q6AY17	Protein ABHD17B	1.51E-02	3.04	Down
6	P10719	ATP synthase subunit beta, mitochondrial	1.57E-02	31.84	Down
7	Q63639	Retinal dehydrogenase 2	1.78E-02	12.13	Down
8	Q9QX79	Fetuin-B	2.82E-02	253.41	Down
9	Q29RW1	Myosin-4	3.51E-02	1.52	Down
10	P02093	Hemoglobin subunit beta	3.79E-04	Infinity	UP
11	P31977	Ezrin	4.01E-04	Infinity	UP
12	Q63342	Dimethylglycine dehydrogenase, mitochondrial	4.44E-04	11.46	UP
13	Q561S0	NADH dehydrogenase [ubiquinone] 1 alpha subcomplex subunit 10, mitochondrial	6.69E-04	Infinity	UP
14	P21575	Dynamin-1	6.90E-04	Infinity	UP
15	O35244	Peroxiredoxin-6	1.02E-03	9.41	UP
16	Q7TP54	Rho family-interacting cell polarization regulator 2	1.66E-03	367.84	UP
17	Q6UPE1	Electron transfer flavoprotein-ubiquinone oxidoreductase, mitochondrial	1.85E-03	236.12	UP
18	P20059	Hemopexin	2.71E-03	779.02	UP
19	P08011	Microsomal glutathione S-transferase 1	4.90E-03	2.22	UP
20	P21775	3-ketoacyl-CoA thiolase A, peroxisomal	5.79E-03	1.92	UP
21	P52873	Pyruvate carboxylase, mitochondrial	6.01E-03	4.34	UP
22	Q8VHF5	Citrate synthase, mitochondrial	7.10E-03	2.54	UP
23	Q68FT3	Pyridine nucleotide-disulfide oxidoreductase domain-containing protein 2	7.26E-03	2.20	UP
24	P38983	40S ribosomal protein SA	7.35E-03	Infinity	UP
25	Q99NA5	Isocitrate dehydrogenase [NAD] subunit alpha, mitochondrial	8.96E-03	378.68	UP
26	Q4FZR5	Adrenocortical dysplasia protein homolog	8.96E-03	Infinity	UP
27	P07633	Propionyl-CoA carboxylase beta chain, mitochondrial	9.21E-03	Infinity	UP
28	P05182	Cytochrome P450 2E1	9.75E-03	1.66	UP
29	Q9Z1P2	Alpha-actinin-1	1.04E-02	1.63	UP
30	P11598	Protein disulfide-isomerase A3	1.68E-02	Infinity	UP
31	A0JPQ4	Tripartite motif-containing protein 72	1.74E-02	6.99	UP
32	Q6IFX1	Keratin, type I cytoskeletal 24	1.75E-02	2.34	UP
33	P63102	14-3-3 protein zeta/delta	2.26E-02	9.12	UP
34	P62755	40S ribosomal protein S6	2.46E-02	11.25	UP
35	P63018	Heat shock cognate 71 kDa protein	3.01E-02	1.81	UP
36	P13676	Acylamino-acid-releasing enzyme	3.24E-02	Infinity	UP

37	Q6UPE0	Choline dehydrogenase, mitochondrial	4.11E-02	Infinity	UP
38	Q9Z270	Vesicle-associated membrane protein-associated protein A	4.59E-02	Infinity	UP
39	Q6P7D4	Cytochrome P450 20A1	4.85E-02	Infinity	UP

	LIVER				
1.	O08839	Bridging integrator 1	6.87E-04	54.51	Down
2.	O70453	Heme oxygenase (decycling) 2, pseudogene 1	2.59E-04	44.96	Down
3.	P01161	Natriuretic peptide A	2.10E-05	42.90	Down
4.	Q9QZ76	Myoglobin	1.26E-05	35.49	Down
5.	P03957	Matrix metallopeptidase 3	4.67E-06	33.16	Down
6.	Q9Z1P2	Actinin alpha 1	1.38E-07	30.77	Down
7.	P37136	Acetylcholinesterase (Cartwright blood group)	3.79E-04	30.68	Down
8.	P02563	Myosin heavy chain 6	7.88E-06	27.98	Down
9.	P04692	Tropomyosin 1, alpha	7.03E-05	24.37	Down
10.	P23693	Troponin I3, cardiac type	1.57E-04	23.21	Down
11.	P62632	Eukaryotic translation elongation factor 1 alpha 2	4.56E-05	18.99	Down
12.	Q5BK63	NADH:ubiquinone oxidoreductase subunit A9	1.11E-02	16.32	Down
13.	P04276	GC, vitamin D binding protein	1.04E-04	12.88	Down
14.	P50753	Troponin T2, cardiac type	2.79E-05	12.54	Down
15.	P02564	Myosin heavy chain 7	1.26E-04	10.56	Down
16.	P47858	Phosphofructokinase, muscle	2.44E-04	10.18	Down
17.	Q68FT1	Coenzyme Q9	1.07E-02	9.69	Down
18.	Q7TP90	Arrestin domain containing 4	1.01E-02	9.37	Down
19.	P62882	G protein subunit beta 5	5.76E-07	8.23	Down
20.	P16409	Myosin light chain 3	1.60E-06	7.91	Down
21.	Q9JHZ4	GRIP1 associated protein 1	2.90E-05	7.19	Down
22.	P07483	Fatty acid binding protein 3	1.35E-05	7.10	Down
23.	P20788	Ubiquinol-cytochrome c reductase, Rieske iron-sulfur polypeptide 1	2.44E-03	7.03	Down
24.	P26284	Pyruvate dehydrogenase E1 alpha 1 subunit	5.42E-04	6.35	Down
25.	P09634	Homeobox A7	1.46E-02	6.16	Down
26.	P12075	Cytochrome c oxidase subunit 5B	6.75E-04	5.17	Down
27.	P24090	Alpha 2-HS glycoprotein	3.65E-03	4.94	Down
28.	P42123	Lactate dehydrogenase B	3.07E-04	4.78	Down
29.	P09605	Creatine kinase, mitochondrial 2	3.82E-05	4.68	Down
30.	P70580	Progesterone receptor membrane component 1	4.86E-02	4.65	Down
31.	P11980	Pyruvate kinase M1/2	3.53E-04	4.52	Down
32.	P08733	Myosin light chain 2	2.17E-05	4.39	Down
33.	Q9Z244	Guanosine monophosphate reductase	3.39E-04	3.80	Down
34.	Q5I0E8	Kinesin family member 22	3.68E-02	3.68	Down
35.	P23928	Crystallin alpha B	4.07E-08	3.65	Down
36.	P11240	Cytochrome c oxidase subunit 5A	1.21E-03	3.64	Down
37.	Q62920	PDZ and LIM domain 5	3.86E-03	3.42	Down
38.	Q9WUH4	Four and a half LIM domains 1	5.74E-05	3.37	Down
39.	Q62658	FK506 binding protein 1A	7.81E-04	3.36	Down
40.	P05065	Aldolase, fructose-bisphosphate A	9.36E-06	3.29	Down
41.	P62898	Cytochrome c, somatic	1.26E-03	3.18	Down
42.	Q5XIE6	3-hydroxyisobutyryl-coa hydrolase	2.44E-03	3.18	Down
43.	Q6AYN4	Phytanoyl-coa 2-hydroxylase interacting protein like	2.93E-02	3.17	Down
44.	Q9ER34	Aconitase 2	3.85E-04	3.16	Down
45.	P14046	Murinoglobulin 1	1.82E-03	2.99	Down

46.	P19234	NADH:ubiquinone oxidoreductase core subunit V2	2.58E-03	2.98	Down
47.	P68511	Tyrosine 3-monooxygenase/tryptophan 5-monooxygenase activation protein eta	1.09E-02	2.92	Down
48.	Q6AY17	Abhydrolase domain containing 17B	2.41E-03	2.87	Down
49.	Q6AYT0	Crystallin zeta	1.06E-02	2.78	Down
50.	O35115	Four and a half LIM domains 2	3.84E-02	2.70	Down
51.	Q99NA5	Isocitrate dehydrogenase 3 (NAD(+)) alpha	4.33E-04	2.66	Down
52.	P85972	Vinculin	1.27E-03	2.65	Down
53.	P97584	Prostaglandin reductase 1	1.34E-02	2.56	Down
54.	P58775	Tropomyosin 2, beta	1.10E-02	2.53	Down
55.	P17425	3-hydroxy-3-methylglutaryl-coa synthase 1	7.34E-04	2.46	Down
56.	B0BNE5	Esterase D	1.21E-02	2.44	Down
57.	Q4FZR5	ACD, shelterin complex subunit and telomerase recruitment factor	4.30E-02	2.43	Down
58.	P00564	Creatine kinase, M-type	1.99E-04	2.36	Down
59.	P13108	Cytochrome P450, family 2, subfamily d, polypeptide 22	1.29E-03	2.35	Down
60.	P31000	Vimentin	2.52E-03	2.30	Down
61.	Q6IG12	Keratin 7	1.37E-03	2.29	Down
62.	P16638	ATP citrate lyase	3.49E-03	2.25	Down
63.	P53534	Glycogen phosphorylase B	8.81E-04	2.22	Down
64.	P19643	Monoamine oxidase B	2.42E-04	2.20	Down
65.	O55171	Acyl-coa thioesterase 2	4.56E-04	2.13	Down
66.	P14668	Annexin A5	1.24E-02	2.06	Down
67.	P09495	Tropomyosin 4	4.72E-02	2.05	Down
68.	Q8VHF5	Citrate synthase	5.84E-04	1.99	Down
69.	P13413	Troponin I1, slow skeletal type	3.80E-04	1.99	Down
70.	P10867	Gulonolactone (L-) oxidase	3.71E-03	1.97	Down
71.	Q6IFX1	Keratin 24	2.48E-03	1.94	Down
72.	P19488	UDP glucuronosyltransferase family 2 member B15	2.82E-04	1.91	Down
73.	Q8CG45	Aldo-keto reductase family 7 member A2	6.84E-05	1.91	Down
74.	P17209	Myosin light chain 4	1.99E-02	1.90	Down
75.	P09875	UDP glucuronosyltransferase family 2 member B17	2.77E-02	1.88	Down
76.	Q7TP54	RHO family interacting cell polarization regulator 2	3.99E-02	1.87	Down
77.	Q64565	Alanine--glyoxylate aminotransferase 2	1.83E-03	1.86	Down
78.	Q05962	Solute carrier family 25 member 4	5.72E-04	1.85	Down
79.	Q09073	Solute carrier family 25 member 5	5.72E-04	1.85	Down
80.	P19633	Calsequestrin 1	3.64E-02	1.80	Down
81.	P62828	RAN, member RAS oncogene family	4.25E-03	1.80	Down
82.	P70470	Lysophospholipase I	2.91E-02	1.79	Down
83.	Q8VIH3	Solute carrier family 28 member 3	3.14E-03	1.78	Down
84.	Q06647	ATP synthase peripheral stalk subunit OSCP	3.38E-02	1.76	Down
85.	P29418	ATP synthase, H+ transporting, mitochondrial F1 complex, epsilon subunit	3.14E-02	1.76	Down
86.	P02600	Myosin light chain 1	1.05E-04	1.74	Down
87.	P12346	Transferrin	3.77E-04	1.72	Down
88.	Q5HZY2	Secretion associated Ras related gtpase 1B	4.33E-02	1.71	Down
89.	Q5M9I5	Ubiquinol-cytochrome c reductase hinge protein like	3.03E-02	1.67	Down
90.	P06761	Heat shock protein family A (Hsp70) member 5	1.10E-02	1.66	Down
91.	Q63108	Carboxylesterase 1E	9.86E-03	1.66	Down
92.	P56571	Glutamine amidotransferase like class 1 domain containing 3A	8.95E-04	1.65	Down
93.	P09812	Glycogen phosphorylase, muscle associated	2.26E-02	1.65	Down
94.	P49432	Pyruvate dehydrogenase E1 beta subunit	9.12E-05	1.61	Down
95.	P08009	Glutathione S-transferase mu 2	1.95E-04	1.59	Down
96.	P62703	Ribosomal protein S4 Y-linked 1	3.72E-02	1.58	Down

97.	Q66HF1	NADH:ubiquinone oxidoreductase core subunit S1	1.99E-02	1.55	Down
98.	Q8CHM7	2-hydroxyacyl-coa lyase 1	1.07E-02	1.52	Down
99.	Q4G064	Coenzyme Q5, methyltransferase	2.46E-02	1.51	UP
100.	P50463	Cysteine and glycine rich protein 3	2.83E-06	1.54	UP
101.	Q66HS7	PDZ and LIM domain 3	7.96E-09	1.58	UP
102.	Q5I0P2	Glycine cleavage system protein H	4.79E-02	1.51	UP
103.	P31210	Aldo-keto reductase family 1 member D1	2.49E-02	1.51	UP
104.	P18886	Carnitine palmitoyltransferase 2	1.24E-02	1.51	UP
105.	Q6AYQ2	Aldo-keto reductase family 1 member C2	9.61E-03	1.51	UP
106.	P57113	Glutathione S-transferase zeta 1	8.24E-03	1.52	UP
107.	P05179	Cytochrome P450 family 2 subfamily C member 8	1.11E-02	1.53	UP
108.	Q9WVK7	Hydroxyacyl-coa dehydrogenase	1.36E-02	1.53	UP
109.	Q498T9	Leucine rich repeat containing 8 VRAC subunit C	4.37E-02	1.54	UP
110.	P0C2X9	Aldehyde dehydrogenase 4 family member A1	3.98E-02	1.54	UP
111.	P62250	Ribosomal protein S16	4.57E-02	1.54	UP
112.	O70257	Syntaxin 7	3.57E-02	1.55	UP
113.	P04903	Glutathione S-transferase alpha 5	4.80E-04	1.56	UP
114.	P15149	Cytochrome P450, family 2, subfamily a, polypeptide 12	1.12E-02	1.57	UP
115.	P35435	ATP synthase F1 subunit gamma	9.66E-04	1.57	UP
116.	Q8VID1	Dehydrogenase/reductase 4	2.11E-02	1.58	UP
117.	P32755	4-hydroxyphenylpyruvate dioxygenase	6.24E-03	1.59	UP
118.	P04785	Prolyl 4-hydroxylase subunit beta	1.05E-02	1.59	UP
119.	P07896	Enoyl-coa hydratase and 3-hydroxyacyl coa dehydrogenase	3.68E-02	1.59	UP
120.	P62963	Profilin 1	4.86E-02	1.59	UP
121.	P00388	Cytochrome p450 oxidoreductase	2.46E-03	1.61	UP
122.	P38659	Protein disulfide isomerase family A member 4	2.36E-02	1.61	UP
123.	P14408	Fumarate hydratase	3.31E-03	1.62	UP
124.	Q66HC9	Dynein axonemal intermediate chain 2	8.70E-03	1.62	UP
125.	P08430	UDP glucuronosyltransferase family 1 member A6	3.19E-03	1.62	UP
126.	P02692	Fatty acid binding protein 1	1.02E-03	1.63	UP
127.	P04462	Myosin heavy chain 8	1.99E-04	1.63	UP
128.	P36511	UDP glucuronosyltransferase family 2 member B7	2.35E-02	1.63	UP
129.	Q02974	Ketohexokinase	3.43E-02	1.63	UP
130.	P62738	Actin, alpha 2, smooth muscle, aorta	2.43E-02	1.63	UP
131.	Q68FT5	Betaine--homocysteine S-methyltransferase 2	2.75E-02	1.64	UP
132.	P00884	Aldolase, fructose-bisphosphate B	5.88E-03	1.65	UP
133.	P07943	Aldo-keto reductase family 1 member B	1.32E-04	1.65	UP
134.	O88267	Acyl-coa thioesterase 1	2.40E-02	1.66	UP
135.	Q66HD0	Heat shock protein 90 beta family member 1	2.49E-02	1.67	UP
136.	P28492	Glutaminase 2	7.99E-05	1.68	UP
137.	Q6P9T8	Tubulin beta 4B class ivb	4.37E-02	1.70	UP
138.	P28037	Aldehyde dehydrogenase 1 family member L1	3.40E-03	1.70	UP
139.	P10860	Glutamate dehydrogenase 1	1.32E-03	1.71	UP
140.	Q9Z1B2	Glutathione S-transferase mu 3	1.57E-02	1.72	UP
141.	P09034	Argininosuccinate synthase 1	1.29E-03	1.75	UP
142.	P07756	Carbamoyl-phosphate synthase 1	4.51E-02	1.75	UP
143.	P10633	Cytochrome P450, family 2, subfamily d, polypeptide 1	2.55E-03	1.75	UP
144.	Q03346	Peptidase, mitochondrial processing beta subunit	2.90E-02	1.75	UP
145.	P11345	Raf-1 proto-oncogene, serine/threonine kinase	1.25E-02	1.76	UP
146.	P12847	Myosin heavy chain 3	5.98E-05	1.77	UP
147.	Q6AXY0	Glutathione S-transferase alpha 2	1.06E-03	1.77	UP

148.	Q68FY0	Ubiquinol-cytochrome c reductase core protein 1	1.99E-02	1.78	UP
149.	Q03248	Beta-ureidopropionase 1	7.13E-05	1.79	UP
150.	P35738	Branched chain keto acid dehydrogenase E1 subunit beta	3.98E-02	1.79	UP
151.	P51635	Aldo-keto reductase family 1 member A1	4.41E-02	1.80	UP
152.	P62083	Ribosomal protein S7	2.00E-04	1.81	UP
153.	P17988	Sulfotransferase family 1A, phenol-preferring, member 1	1.25E-02	1.82	UP
154.	P35859	Insulin like growth factor binding protein acid labile subunit	3.28E-02	1.82	UP
155.	P08541	UDP glucuronosyltransferase family 2 member B28	1.01E-02	1.84	UP
156.	P23457	Aldo-keto reductase family 1, member C14	2.52E-02	1.85	UP
157.	Q71UF4	RB binding protein 7, chromatin remodeling factor	6.08E-03	1.86	UP
158.	P97562	Acyl-coa oxidase 2	1.02E-03	1.86	UP
159.	P62832	Ribosomal protein L23	7.38E-03	1.86	UP
160.	Q5M9H0	Ankyrin repeat and sterile alpha motif domain containing 3	3.55E-02	1.88	UP
161.	Q9ES39	Nude neurodevelopment protein 1	1.67E-04	1.88	UP
162.	B0BNN3	Carbonic anhydrase 1	6.75E-03	1.89	UP
163.	Q63276	Bile acid-coa:amino acid N-acyltransferase	9.36E-03	1.89	UP
164.	P50237	Sulfotransferase family 1C member 3	1.96E-02	1.89	UP
165.	P00684	Ribonuclease A family member 1, pancreatic	6.90E-06	1.90	UP
166.	Q5XIL2	Phosphatidylinositol 4-kinase type 2 beta	4.82E-02	1.90	UP
167.	Q07116	Sulfite oxidase	2.01E-02	1.90	UP
168.	P62630	Eukaryotic translation elongation factor 1 alpha 1	1.73E-02	1.91	UP
169.	P62271	Ribosomal protein S18	4.37E-03	1.93	UP
170.	P62902	Ribosomal protein L31	3.35E-02	1.93	UP
171.	P97521	Solute carrier family 25 member 20	1.39E-03	1.93	UP
172.	Q9JLJ3	Aldehyde dehydrogenase 9 family member A1	3.18E-05	1.96	UP
173.	P22791	3-hydroxy-3-methylglutaryl-coa synthase 2	9.25E-03	1.99	UP
174.	P13444	Methionine adenosyltransferase 1A	3.65E-03	1.99	UP
175.	P25093	Fumarylacetoacetate hydrolase	9.39E-04	2.00	UP
176.	P07308	Stearoyl-coa desaturase	3.97E-02	2.04	UP
177.	O09171	Betaine--homocysteine S-methyltransferase	1.65E-03	2.04	UP
178.	P09811	Glycogen phosphorylase L	4.88E-03	2.04	UP
179.	Q6AYS8	Hydroxysteroid 17-beta dehydrogenase 11	3.17E-03	2.05	UP
180.	P10760	Adenosylhomocysteinase	1.43E-02	2.09	UP
181.	P41123	Ribosomal protein L13	4.80E-02	2.11	UP
182.	Q76MX4	MAF bzip transcription factor G	7.46E-03	2.12	UP
183.	O35077	Glycerol-3-phosphate dehydrogenase 1	3.95E-04	2.13	UP
184.	Q5PPL3	NAD(P) dependent steroid dehydrogenase-like	1.12E-02	2.17	UP
185.	P51652	Aldo-keto reductase family 1 member C3	7.52E-03	2.18	UP
186.	P0DMW0	Heat shock protein family A (Hsp70) member 1A	3.63E-02	2.20	UP
187.	Q68FS4	Leucine aminopeptidase 3	5.39E-04	2.22	UP
188.	P46418	Glutathione S-transferase alpha 3	1.52E-04	2.27	UP
189.	Q63707	Dihydroorotate dehydrogenase (quinone)	4.18E-03	2.28	UP
190.	P51653	Glypican 2	4.23E-02	2.29	UP
191.	Q8R426	Potassium voltage-gated channel interacting protein 1	8.80E-03	2.30	UP
192.	P21396	Monoamine oxidase A	2.28E-03	2.30	UP
193.	P07895	Superoxide dismutase 2	4.23E-06	2.35	UP
194.	Q6P6G5	Yip1 domain family member 1	3.21E-02	2.36	UP
195.	P16036	Solute carrier family 25 member 3	7.42E-05	2.37	UP
196.	P07872	Acyl-coa oxidase 1	1.40E-04	2.39	UP
197.	Q63041	PZP, alpha-2-macroglobulin like	6.01E-03	2.39	UP
198.	P50398	GDP dissociation inhibitor 1	2.94E-04	2.39	UP

199.	Q4KLZ6	Triokinase and FMN cyclase	2.81E-03	2.40	UP
200.	Q5XIJ8	Coiled-coil domain containing 65	1.40E-02	2.42	UP
201.	O70142	SHC adaptor protein 2	5.44E-03	2.50	UP
202.	Q561R7	General transcription factor IIH subunit 3	1.31E-02	2.51	UP
203.	P04904	Glutathione S-transferase alpha 1	3.21E-05	2.51	UP
204.	Q5XIH4	Lipoic acid synthetase	3.75E-03	2.60	UP
205.	P05178	Cytochrome P450 family 2 subfamily C member 19	5.27E-04	2.64	UP
206.	A0JPI9	Leucine rich repeat containing 74A	2.78E-04	2.66	UP
207.	P70473	Alpha-methylacyl-coa racemase	9.72E-04	2.69	UP
208.	P19112	Fructose-bisphosphatase 1	9.32E-05	2.74	UP
209.	P52873	Pyruvate carboxylase	7.44E-03	2.74	UP
210.	Q63610	Tropomyosin 3	1.31E-02	2.75	UP
211.	O08700	Vacuolar protein sorting 45 homolog	4.38E-03	2.82	UP
212.	P09606	Glutamate-ammonia ligase	2.58E-03	2.83	UP
213.	Q9ES08	Potassium two pore domain channel subfamily K member 9	1.82E-02	2.86	UP
214.	P82995	Heat shock protein 90 alpha family class A member 1	1.66E-03	2.89	UP
215.	Q9Z158	Syntaxin 17	1.90E-03	3.07	UP
216.	P01048	Kininogen 2	1.07E-02	3.25	UP
217.	P62425	Ribosomal protein l7a	6.37E-05	3.26	UP
218.	P13255	Glycine N-methyltransferase	5.49E-04	3.33	UP
219.	Q5FVR5	Acyl-coenzyme A amino acid N-acyltransferase 2	6.21E-04	3.34	UP
220.	Q4QQS8	Nucleoporin 85	4.31E-03	3.53	UP
221.	Q63279	Keratin 19	8.30E-06	3.73	UP
222.	P14200	Pro-melanin concentrating hormone	3.33E-02	3.85	UP
223.	P04166	Cytochrome b5 type B	3.66E-05	4.05	UP
224.	Q9ESV6	Glyceraldehyde-3-phosphate dehydrogenase, spermatogenic	2.74E-05	4.31	UP
225.	P11960	Branched chain keto acid dehydrogenase E1, alpha polypeptide	2.51E-05	5.10	UP
226.	Q63250	Forkhead box E3	2.95E-04	6.38	UP
227.	Q68FX6	Calcium binding protein, spermatid associated 1	9.20E-05	8.01	UP
228.	P52847	Sulfotransferase family 1B member 1	1.25E-06	8.42	UP

Kidney					
1.	P21571	ATP synthase peripheral stalk subunit F6	1.72E-02	6.448	Down
2.	Q63633	Solute carrier family 12 member 5	1.08E-02	3.341	Down
3.	Q794F9	4F2 cell-surface antigen heavy chain	7.85E-04	3.324	Down
4.	P22072	Hydroxy-delta-5-steroid dehydrogenase, 3 beta- and steroid delta-isomerase 2	1.60E-02	2.294	Down
5.	P27364	NADPH-dependent 3-keto-steroid reductase Hsd3b5	4.15E-03	2.242	Down
6.	Q920P0	Dicarbonyl and L-xylulose reductase	2.06E-02	2.187	Down
7.	Q6AXV4	SAMM50 sorting and assembly machinery component	1.03E-03	1.915	Down
8.	P97943	Scavenger receptor class B member 1	2.52E-03	1.861	Down
9.	Q63610	Tropomyosin 3	5.97E-03	1.835	Down
10.	P49432	Pyruvate dehydrogenase E1 beta subunit	2.37E-02	1.804	Down
11.	Q5XI32	Capping actin protein of muscle Z-line subunit beta	2.09E-02	1.732	Down
12.	Q920J3	Coronin 6	1.73E-02	1.579	Down
13.	P20817	Cytochrome P450, family 4, subfamily a, polypeptide 14	3.08E-03	1.539	Down
14.	Q63666	Vesicle-associated membrane protein 1	3.03E-02	1.512	Down
15.	P50503	ST13, Hsp70 interacting protein	8.18E-03	1.501	Down
16.	Q8R431	Monoglyceride lipase	8.49E-03	1.556	Up
17.	Q9Z339	Glutathione S-transferase omega 1	8.11E-04	1.606	Up
18.	Q5M7T9	Threonine synthase like 2	3.77E-02	1.618	Up

19.	P00481	Ornithine carbamoyltransferase	4.76E-02	1.621	Up
20.	Q925B5	Synaptotagmin 13	3.64E-03	1.631	Up
21.	P38718	Mitochondrial pyruvate carrier 2	8.05E-03	1.654	Up
22.	P08009	Glutathione S-transferase mu 2	1.03E-02	1.754	Up
23.	P62824	RAB3C, member RAS oncogene family	9.96E-03	1.76	Up
24.	B0BNA5	Coactosin like F-actin binding protein 1	1.40E-03	1.766	Up
25.	P47853	Biglycan	4.44E-02	1.812	Up
26.	P04550	Parathymosin	1.04E-02	1.892	Up
27.	P97636	Interleukin 18	2.18E-02	1.927	Up
28.	P02563	Myosin heavy chain 6	3.59E-02	1.935	Up
29.	P63012	RAB3A, member RAS oncogene family	2.91E-02	1.944	Up
30.	P04916	Retinol binding protein 4	1.10E-02	1.973	Up
31.	P20761	Immunoglobulin heavy chain 6	5.85E-03	2.015	Up
32.	Q9JM01	Cyclin dependent kinase like 3	3.50E-02	2.143	Up
33.	P08010	Glutathione S-transferase mu 1	1.76E-02	2.152	Up
34.	Q9ESV6	Glyceraldehyde-3-phosphate dehydrogenase, spermatogenic	2.69E-02	2.165	Up
35.	P47875	Cysteine and glycine rich protein 1	2.38E-03	2.236	Up
36.	P46844	Biliverdin reductase A	1.64E-02	2.251	Up
37.	Q5I0C3	Methylcrotonoyl-coa carboxylase 1	4.16E-03	2.287	Up
38.	P47861	Synaptotagmin 5	4.48E-02	2.293	Up
39.	Q66HF8	Aldehyde dehydrogenase 1 family member B1	1.36E-02	2.39	Up
40.	Q6AYP3	Tetratricopeptide repeat domain 29	2.90E-03	2.394	Up
41.	P11980	Pyruvate kinase M1/2	1.61E-02	2.556	Up
42.	Q5PQN0	Neurocalcin delta	2.98E-03	2.662	Up
43.	P02680	Fibrinogen gamma chain	4.65E-02	3.082	Up
44.	B9TQX4	Upper zone of growth plate and cartilage matrix associated	1.49E-02	3.892	Up
45.	O89049	Thioredoxin reductase 1	2.50E-03	4.011	Up
46.	P00697	Lysozyme	7.04E-04	4.372	Up
47.	P24090	Alpha 2-HS glycoprotein	6.28E-03	4.52	Up
48.	P02692	Fatty acid binding protein 1	4.18E-02	9.349	Up
49.	Q63941	RAB3B, member RAS oncogene family	2.91E-02	9.755	Up
50.	P62907	Ribosomal protein l10a	1.63E-02	25.781	Up
51.	P23785	Granulin precursor	6.80E-03	102.361	Up
52.	Q9R1J4	Myocilin	1.49E-03	319.242	Up
