# Supplementary material for: Proteomic Analysis of Morphologically Changed Tissues after Prolonged Dexamethasone Treatment
Source: Int J Mol Sci. 2019 Jun 26;20(13):3122. doi: 10.3390/ijms20133122 (PMC6650964; doi:10.3390/ijms20133122)
Supplement: Supplementary file 1 [file ijms-20-03122-s001.zip › Table S 4..pdf]

**Table S 4**	Canonical Pathways	-log(p-value)	Ratio	Number of molecules	Total number of molecules
Overall					
1	LPS/IL-1 Mediated Inhibition of LXR and RXR Function	21.2	0.17	38	224
2	Xenobiotic Metabolism Signaling	20.8	0.142	43	303
3	Serotonin Degradation	16.8	0.284	21	74
4	NRF2-mediated Oxidative Stress Response	14.9	0.146	30	206
5	Mitochondrial Dysfunction	14.4	0.158	27	171
6	Dopamine Degradation	11.9	0.351	13	37
Brain					
1	Leucine Degradation I	6.27	0.444	11	206
2	NRF2-mediated Oxidative Stress Response	5.93	0.053 4	5	24
3	Glutathione Redox Reactions I	5.87	0.208	12	292
4	Sirtuin Signaling Pathway	5.23	0.041 1	8	148
5	Phagosome Maturation	4.49	0.054 1	4	26
6	Glycolysis I	4.24	0.154	4	26
Heart					
1	LPS/IL-1 Mediated Inhibition of LXR and RXR Function	9.02	0.067 6		
2	NRF2-mediated Oxidative Stress Response	8.85	0.072 5		
3	Xenobiotic Metabolism Signaling	8.31	0.055 2		
4	Glutathione-mediated Detoxification	8.16	0.226		
5	Serotonin Degradation	7.68	0.117		
6	Glutathione Redox Reactions I	5.78	0.208		
Muscle					

1	Glutathione Redox Reactions I	3.01	0.087	2	23
2	TCA Cycle II (Eukaryotic)	3.01	0.087	2	23
3	p70S6K Signaling	2.65	0.023<br>3	3	129
4	Guanine and Guanosine Salvage I	2.39	0.5	1	2
5	Choline Degradation I	2.39	0.5	1	2
6	Regulation of Cellular Mechanics by Calpain Protease	2.26	0.036<br>4	2	55
Liver					
1	LPS/IL-1 Mediated Inhibition of LXR and RXR Function	16.1	0.104	15	77
2	Xenobiotic Metabolism Signaling	15.5	0.086<br>2	13	70
3	Serotonin Degradation	14.8	0.195	17	171
4	Superpathway of Melatonin Degradation	12.6	0.186	11	65
5	Mitochondrial Dysfunction	11.7	0.099<br>4	8	37
6	Melatonin Degradation I	10.3	0.169	11	109
Kidney					
1	LPS/IL-1 Mediated Inhibition of LXR and RXR Function	7.32	0.037<br>2	8	215
2	Glutathione-mediated Detoxification	4.27	0.1	3	30
3	NRF2-mediated Oxidative Stress Response	4.06	0.027	5	185
4	LXR/RXR Activation	3.69	0.033<br>6	4	119
5	FXR/RXR Activation	3.61	0.032	4	125
6	Mineralocorticoid Biosynthesis	3.5	0.182	2	11
