# Supplementary material for: Proteomic Analysis of Morphologically Changed Tissues after Prolonged Dexamethasone Treatment
Source: Int J Mol Sci. 2019 Jun 26;20(13):3122. doi: 10.3390/ijms20133122 (PMC6650964; doi:10.3390/ijms20133122)
Supplement: Supplementary file 1 [file ijms-20-03122-s001.zip › Table S3 .pdf]

**Table S3**	Name	p-value range	# Molecules				
**Brain**							
1	Cellular Compromise	4.22E-03 - 1.57E-10	42	ALDOC<br>AP2A2<br>ATP1B2<br>ATP6V0A1<br>C3<br>CES1<br>COTL1<br>CP<br>CTSC<br>GPI<br>GRN	GSTP1<br>GZMB<br>HP<br>HPX<br>IDH1<br>KRT18<br>MAPT<br>MECP2<br>MGST1<br>MOG<br>MSN	NEFL<br>Nefm<br>PAFAH1B2<br>PDXK<br>PLP1<br>PNP<br>PPP2CA<br>PPT1<br>PRDX6<br>PSMB1<br>PYGL	RAB3A<br>RAB7A<br>RALA<br>RAP1B<br>RNLS<br>SLC1A3<br>STAMBP<br>TFAM<br>VCP
2	Molecular Transport	5.37E-03 - 2.37E-09	65	ACADM<br>AKR1B1<br>ALDH1A1<br>AP2A2<br>AP2M1<br>ATP1B2<br>ATP2B2<br>ATP5F1B<br>ATP6V0A1<br>BCAT2<br>C3<br>CA1<br>CACNA2D3<br>CES1<br>CLTRN<br>CP<br>CPLX1	DBI<br>DDX39B<br>EIF2D<br>EPHX1<br>GAD2<br>GAPDH<br>GAPDHS<br>GGT1<br>GSR<br>H2AFY<br>HADH<br>HNRNPA2B1<br>HP<br>HPX<br>IDH1<br>LETM1<br>LMNA	LRRC8A<br>LRRC8C<br>MAPT<br>MECP2<br>MSN<br>NAPA<br>PCK1<br>PDCD6<br>PHAX<br>PHB<br>PNP<br>PPT1<br>PRDX2<br>PRDX6<br>RAB34<br>RAB3A<br>RAB3C	RAB4A<br>RAB7A<br>RALA<br>S100B<br>SCARB1<br>SEC22B<br>SLC1A3<br>SLC25A4<br>STX1A<br>SV2A<br>SYN2<br>TOMM40<br>TPT1<br>VPS45
3	Cell Morphology	4.96E-03 - 4.78E-09	51	AP2M1<br>ATP1B2<br>ATP2B2<br>C1QBP<br>C3<br>CACNA2D3	GRN<br>HNRNPA2B1<br>KLK8<br>KRT18<br>LETM1<br>LMNA	NEFL<br>Nefm<br>PCK1<br>PLP1<br>PPP2CA<br>PPT1	S100B<br>SLC1A3<br>STIP1<br>STX1A<br>SV2A<br>SYN2

				CAPZB<br>CEP83<br>CES1<br>CP<br>CPLX1<br>CSRP1<br>GAPDH<br>GDI1	LYPLA1<br>MAPT<br>MECP2<br>MOG<br>MSN<br>MUSK<br>MYBPC3<br>MYH6	RAB35<br>RAB3A<br>RAB4A<br>RAB7A<br>RALA<br>RAP1B<br>RIPOR2<br>RPS14	TFAM<br>VCP<br>Wasl
**Heart**							
1	Amino Acid Metabolism	4.25E-03 - 1.29E-12	21	ARG1<br>ASPA<br>ASS1<br>BAAT<br>BHMT<br>BHMT2	CKMT2<br>CPS1<br>CTH<br>DDAH2<br>FABP3<br>GLYAT	Gnmt<br>GSN<br>GSTZ1<br>MAT1A<br>NIT2<br>STX1A	SYN1<br>SYN2<br>UPB1
2	Small Molecule Biochemistry	7.96E-03 - 1.29E-12	86	A2M<br>ABCC2<br>ACAA2<br>Acot1<br>ACOT7<br>ACOX1<br>ADH1C<br>Akr1c14<br>AKR7A3<br>Aldh1a7<br>ALDOB<br>AMACR<br>ANXA1<br>ARG1<br>ASPA<br>ASS1<br>ATP2B2<br>ATP5F1D<br>ATP5PF<br>ATP5PO<br>BAAT<br>BDH2	BHMT<br>BHMT2<br>CALCB<br>Calm1<br>CAT<br>CES1<br>CISD1<br>CKMT2<br>CMPK1<br>CPLX2<br>CPS1<br>CSRP3<br>CTH<br>CYB5A<br>Cyp2c23<br>CYP2C9<br>CYP4A22<br>DAB2<br>DBI<br>DDAH2<br>DECR2<br>DPYS	EPHX1<br>FABP1<br>FABP3<br>FBP1<br>GLYAT<br>GMFB<br>Gnmt<br>GPD1<br>GSN<br>GSTM1<br>GSTM2<br>GSTM5<br>GSTZ1<br>HSD11B1<br>Hsd3b4<br>HSPA5<br>IDH1<br>MAPK1<br>MAT1A<br>MIF<br>MYH9<br>NAPRT	NIT2<br>OCRL<br>PAFAH1B2<br>PGM1<br>PGR<br>PLCB1<br>PNP<br>RALA<br>RGN<br>RPSA<br>SACM1L<br>SNCG<br>SOD1<br>SORD<br>STX1A<br>Sult1a1<br>SYN1<br>SYN2<br>UGT2B15<br>UPB1
3	Nucleic Acid Metabolism	6.42E-03 - 1.33E-10	32	ACAA2<br>Acot1	ATP5PF<br>ATP5PO	CES1<br>CMPK1	GLYAT<br>Gnmt

				ACOT7	BAAT	CYP2C9	GPD1
				ACOX1	BHMT	DBI	MAPK1
				ALDOB	BHMT2	DECR2	MAT1A
				AMACR	CALCB	DPYS	NAPRT
				ATP2B2	Calm1	FABP1	PNP
				ATP5F1D	CAP1	GDA	SOD1
**Muscle**							
**1**	Vitamin and Mineral Metabolism	2.00E-02 - 1.01E-04	5	ACAA1	CYP2E1	EZR	HSPA8
				ALDH1A2			
**2**	Energy Production	4.35E-02 - 4.10E-04	5	ALDH1A2	CHDH	CYP2E1	PC
				ACAA1			
**3**	Post-Translational Modification	2.99E-02 - 6.64E-04	5	HSPA8	PDIA3	PRDX6	YWHAZ
				OAT			
**Liver**							
**1**	Amino Acid Metabolism	6.09E-04 - 2.91E-18	26	AGXT2	BHMT	GCSH	HPD
				AHCY	BHMT2	GLS2	MAT1A
				ALDH1L1	CKM	GLUD1	NPPA
				ASS1	CKMT2	GLUL	PKM
				BAAT	CPS1	Gnmt	UPB1
				BCKDHA	CS	GSTZ1	
				BCKDHB	FAH	HADH	
**2**	Small Molecule Biochemistry	6.82E-04 - 2.91E-18	103	ACHE	BAAT	GLUD1	NPPA
				ACLY	BCKDHA	GLUL	NSDHL
				Acnat1/Acnat2	BCKDHB	Gnmt	PC
				ACO2	BHMT	GPD1	PDHA1
				Acot1	BHMT2	GSTA2	PDHB
				ACOT2	CKM	GSTA3	PGRMC1
				ACOX1	CKMT2	GSTZ1	PKM
				ACOX2	CPS1	Gulo	PMCH
				AGXT2	CPT2	HACL1	POR
				AHCY	CS	HADH	PTGR1
				AKR1A1	CYB5B	HMGCS2	SCD
				AKR1B1	CYCS	HPD	SLC25A5
				AKR1C1/AKR1C2	CYP2C19	HSD17B11	SOD2
				Akr1c14	CYP2C8	HSPA1A/HSPA1B	Sult1a1
				AKR1C3	Cyp2d22	IDH3A	SULT1B1

				AKR1D1<br>ALDH1L1<br>ALDH9A1<br>ALDOA<br>ALDOB<br>AMACR<br>ANXA5<br>ASS1<br>Atp5e<br>ATP5F1C<br>ATP5PO	DHRS4<br>EHHADH<br>FABP1<br>FABP3<br>FAH<br>FBP1<br>FH<br>GAPDHS<br>GC<br>GCSH<br>GLS2	KHK<br>MAOA<br>MAOB<br>MAT1A<br>MB<br>MMP3<br>MYH3<br>MYH6<br>MYH7<br>MYH8<br>NDUFS1	TF<br>UGT1A6<br>UGT2B15<br>UGT2B17<br>UGT2B28<br>UGT2B7<br>UPB1<br>UQCRFS1<br>VIM<br>YWHAH
**3**	Lipid Metabolism	6.46E-04 - 2.21E-15	54	ACHE<br>ACLY<br>Acnat1/Acnat2<br>Acot1<br>ACOT2<br>ACOX1<br>ACOX2<br>AKR1A1<br>AKR1B1<br>AKR1C1/AKR1C2<br>Akr1c14<br>AKR1C3<br>AKR1D1<br>AMACR	ANXA5<br>BAAT<br>CPT2<br>CS<br>CYB5B<br>CYCS<br>CYP2C19<br>CYP2C8<br>Cyp2d22<br>DHRS4<br>EHHADH<br>FABP1<br>FABP3<br>FH	GC<br>GSTA2<br>GSTA3<br>HACL1<br>HADH<br>HMGCS2<br>MAOA<br>MB<br>MMP3<br>NPPA<br>NSDHL<br>PDHA1<br>PDHB<br>PGRMC1	PKM<br>POR<br>PTGR1<br>SCD<br>SOD2<br>Sult1a1<br>UGT2B15<br>UGT2B17<br>UGT2B28<br>UGT2B7<br>VIM<br>YWHAH
	**Kidney**						
1	Drug Metabolism	2.18E-02 - 3.88E-06	5	GSTM1<br>GSTM2	GSTO1	SLC3A2	HSD3B2
**2**	Molecular Transport	2.66E-02 - 3.51E-05	16	AHSG<br>BLVRA<br>Cyp4a14<br>FABP1	FGG<br>GAPDHS<br>GRN<br>IL18	MGLL<br>PKM<br>RAB3A<br>RAB3C	RBP4<br>SCARB1<br>SLC12A5<br>SLC3A2
**3**	Small Molecule Biochemistry	2.69E-02 - 3.51E-05 26	26	AHSG<br>ATP5PF<br>BLVRA<br>COTL1<br>Cyp4a14<br>DCXR<br>FABP1	GAPDHS<br>GRN<br>GSTM1<br>GSTM2<br>GSTO1<br>HSD3B2<br>Hsd3b4	IL18<br>LYZ<br>MCCC1<br>MGLL<br>MYH6<br>OTC<br>PKM	RBP4<br>SCARB1<br>SLC12A5<br>SLC3A2<br>TXNRD1
